# Correlating sugar transporter expression and activities to identify transporters for an orphan sugar substrate

**DOI:** 10.1007/s00253-023-12907-4

**Published:** 2024-01-08

**Authors:** Elisabeth Tamayo, Basant Nada, Isabell Hafermann, J. Philipp Benz

**Affiliations:** 1 Fungal Biotechnology in Wood Science, Holzforschung München, TUM School of Life Sciences, Technical University of Munich, Freising, Germany; 2 Faculty of Science, Suez Canal University, Ismailia, Egypt

**Keywords:** *Neurospora crassa*, Sugar transport, Major facilitator superfamily, Carbohydrate metabolism, Lactose

## Abstract

**Abstract:**

Filamentous fungi like *Neurospora crassa* are able to take up and metabolize important sugars present, for example, in agricultural and human food wastes. However, only a fraction of all putative sugar transporters in filamentous fungi has been characterized to date, and for many sugar substrates, the corresponding transporters are unknown. In *N. crassa*, only 14 out of the 42 putative major facilitator superfamily (MFS)–type sugar transporters have been characterized so far. To uncover this hidden potential for biotechnology, it is therefore necessary to find new strategies. By correlation of the uptake profile of sugars of interest after different induction conditions with the expression profiles of all 44 genes encoding predicted sugar transporters in *N. crassa*, together with an exhaustive phylogenetic analysis using sequences of characterized fungal sugar transporters, we aimed to identify transporter candidates for the tested sugars. Following this approach, we found a high correlation of uptake rates and expression strengths for many sugars with dedicated transporters, like galacturonic acid and arabinose, while the correlation is loose for sugars that are transported by several transporters due to functional redundancy. Nevertheless, this combinatorial approach allowed us to elucidate the uptake system for the disaccharide lactose, a by-product of the dairy industry, which consists of the two main cellodextrin transporters CDT-1 and CDT-2 with a minor contribution of the related transporter NCU00809. Moreover, a non-MFS transporter involved in glycerol transport was also identified. Deorphanization of sugar transporters or identification of transporters for orphan sugar substrates by correlation of uptake kinetics with transporter expression and phylogenetic information can thus provide a way to optimize the reuse of food industry by-products and agricultural wastes by filamentous fungi in order to create economic value and reduce their environmental impact.

**Key points:**

• *The *Neurospora crassa* genome contains 30 uncharacterized putative sugar transporter genes.*

• *Correlation of transporter expression and sugar uptake profiles can help to identify transporters for orphan sugar substrates.*

• *CDT-1, CDT-2, and NCU00809 are key players in the transport of the dairy by-product lactose in* N. crassa*.*

**Supplementary Information:**

The online version contains supplementary material available at 10.1007/s00253-023-12907-4.

## Introduction

Filamentous fungi and yeasts have essential roles in carbon turnover during the conversion of complex substrates from nature, like plant biomass. One key step for this is the uptake of sugars across cell membranes, mediated by transmembrane proteins called sugar transporters (Mattam et al. 2022). The diversity of transporters involved in membrane trafficking of sugars has likely evolved from the single function of glucose uptake into the cells (Carbó and Rodríguez 2023). In addition to their great biological importance in cell nutrition and homeostasis, sugar transporters can act as “transceptors” with essential functions for sensing, not only of glucose but also for other sugars such as l-arabinose and cellobiose (Celenza et al. 1988; Madi et al. 1997; Kim et al. 2013; Znameroski et al. 2014; Gu et al. 2023). By their involvement in sugar perception, sugar transporters trigger signaling cascades, leading to the induction of biomass-hydrolyzing enzymes such as cellulases and hemicellulases (Adnan et al. 2017; Wu et al. 2020; Mattam et al. 2022). A complex network of transcription factors and sugar transporters is furthermore involved in the mechanism of carbon catabolite repression (CCR) that regulates the utilization of preferred carbon sources in the cells. Wang et al. (2017) found that the *Neurospora crassa* glucose transporters HGT-1 and HGT-2 contribute to the activation of CCR, including the activation of *cre-1* expression, a key transcription factor in the pathway, which was found to control the expression of many sugar transporters itself (Wu et al. 2020). In general, the expression and activity of transporter proteins are tightly regulated at the transcriptional level, while additional post-translational and endocytosis mechanisms are in place in order to respond rapidly to sugar fluctuations, both during excess and deficiency (Barata-Antunes et al. 2021).

In structural terms, about 99% of the sugar transporters of filamentous fungi belong to the major facilitator superfamily (MFS) (Nogueira et al. 2020) and are included in the Transporter Classification Database (TCDB, Saier et al. 2021), where they are categorized as TC 2.A.1. This superfamily is ubiquitous in all organisms and shows substantial variation of the structural and mechanistic principles between protein members (Quistgaard et al. 2016). Most of them belong to the sugar porter family (TC 2.A.1.1). However, there are also sugar transporters among the drug/H^+^ antiporters (TC 2.A.1.2), which include fructose transporters from *Saccharomycotina* fungi (Leandro et al. 2011; Gonçalves et al. 2016) and the fucose: H^+^ symporters (TC 2.A.1.7), which however also transport other 6-deoxy-hexoses, such as l-rhamnose (Sloothaak et al. 2016; Wu et al. 2020). Putative non-MFS SWEET sugar transporters (TC 2.A.123; Chen et al. 2010) have been found in the genome of chytrid fungi (Hu et al. 2016; Jia et al. 2017). In addition, fungal aquaporins (TC 1.A.8) can transport small uncharged molecules across membranes including polyols (Pettersson et al. 2005). A number of genome-wide identification studies of sugar transporters in fungi have been performed to date, including analyses of genomes from the yeast *Kluyveromyces marxianus* and the saprophytic fungi *Aspergillus niger*, *Aspergillus nidulans*, *Aspergillus oryzae*, and *Trichoderma reesei* (Peng et al. 2018; Varela et al. 2019a; Lv et al. 2020; Nogueira et al. 2020). Nonetheless, although already explored (Li et al. 2014; Gao et al. 2017; Wu et al. 2020), no exhaustive in silico analysis of the complete set of *N. crassa* sugar transporters has been performed so far.

Filamentous fungi are able to take up and metabolize important sugars present in agricultural and human food wastes, including lactose, a disaccharide consisting of galactose and glucose subunits (β-d-galactopyranosyl-(1 → 4)-d-glucose). *N. crassa* is an important reference organism for genetic and biochemical studies and is also able to grow on lactose as sole carbon source. Germinated conidia of *N. crassa* can take up lactose and metabolize it after a few hours (Lester et al. 1962). Early studies showed that the uptake rate of lactose and galactose is lower than of fructose and glucose in *N. crassa*, probably due to the inability to rapidly metabolize these sugars (Schneider and Wiley 1971). While it was observed that CDT-1 can transport lactose in a heterologous system (Liu et al. 2016), the overall lactose transport system in this fungus remains an enigma.

The aim of this study was to identify the sugar transporters of *N. crassa* that are responsible for the uptake of carbohydrate substrates, for which the uptake system is still unknown. We applied gene expression and phylogenetic analyses, which we combined for the first time with the analysis of sugar transport capacities in a range of induction conditions. Doing so, we identified the transporter proteins involved in lactose metabolism in this reference system and were able to correlate the uptake of glycerol, as a central carbohydrate metabolite, with the expression of the aquaporin NCU08052, whose involvement in glycerol transport was verified by the growth and uptake assays with the corresponding mutant. These studies will lay the groundwork for the “deorphanization” of uncharacterized sugar transporters as well as the discovery of new substrates for already characterized sugar transporters in *N. crassa*.

## Material and methods

### Biological material and growth conditions

*N. crassa* strains used in this study are listed in Table 1. Knockout strains were acquired from the Fungal Genetic Stock Center (FGSC; McCluskey et al. 2010). *N. crassa* strains were grown on 2% sucrose Vogel’s minimal medium (Vogel 1956) slants in the dark at 30 °C for 2 days and transferred to light/darkness cycle conditions at 25 °C for conidiation. All assays were done with biological triplicates for each strain per condition.

**Table 1:** *Neurospora crassa* strains used in this work

Strain	Relevant genotype	Reference
WT	Oak Ridge wild type	FGSC # 2489
∆*cdt-1*	Δ*NCU00801::hph*	FGSC # 16575
∆*cdt-2*	Δ*NCU08114::hph*	FGSC # 17869
∆*cdt-1*∆*cdt-2*	Δ*NCU00801::hph*, Δ*NCU08114::hph*	Crossing of FGSC # 16575 and FGSC # 17869
Δ*NCU00809*	Δ*NCU00809::hph*	FGSC # 16396
Δ*NCU08052*	Δ*NCU08052::hph*	FGSC # 12016

*FGSC* Fungal Genetics Stock Center (McCluskey et al. 2010)

### Identification of sugar transporter genes in *N. crassa* genome and sequence analyses

After an extensive literature search for characterized transporters, the amino acid sequences were downloaded from FungiDB (fungidb.org/) or the corresponding freely accessible genome databases (https://www.broadinstitute.org/fungal-genome-initiative; http://www.yeastgenome.org/; http://genome.jgi-psf.org/). Amino acid sequences of *Saccharomyces cerevisiae* sugar transporters were retrieved from the *Saccharomyces* genome database (http://www.yeastgenome.org/). These sequences were used to search for orthologous sequences in the filtered model dataset of *N. crassa* on the JGI website (https://mycocosm.jgi.doe.gov/Neucr2/Neucr2.home.html) using the Basic Local Alignment Search Tool (BLAST) algorithm (Altschul et al. 1990) via a protein BLAST. A second search was performed via a keyword search directly. All amino acid sequences of the characterized fungal sugar transporters from distinct taxonomic groups together with the sugar transporter candidates from *N. crassa* were used in the phylogenetic analysis.

Full-length amino acid sequences were aligned by Clustal Omega (Sievers and Higgins 2018; https://www.ebi.ac.uk/Tools/msa/clustalo/). Alignments were imported into the Molecular Evolutionary Genetics Analysis (MEGA) package version 11 (Tamura et al. 2021). A phylogenetic analysis was conducted by the neighbor-joining (NJ) method, implemented in MEGA, with a pairwise deletion of gaps and the Poisson model for distance calculation. Bootstrap analyses were carried out with 1000 replicates. The evolutionary tree was drawn to scale. Predictions of subcellular localizations were made using WoLF PSORT (https://wolfpsort.hgc.jp). In silico analysis of transmembrane domains of sugar transporters was performed using the server Protter (http://wlab.ethz.ch/protter/#).

### Expression analyses

Data from gene expression profiles of all the identified putative sugar transporters from *N. crassa* from Wu et al. (2020) together with gene expression data of the ten characterized sugar transporters from *A. niger* from Peng et al. (2018) were used. Eighteen induction conditions were selected, including monosaccharide (d-fructose, d-galactose, d-galacturonic acid, d-mannose, d-xylose, l-arabinose, and l-rhamnose), related disaccharides (sucrose and maltose), and complex sugars (arabinan, cellulose, galactan, inulin, pectin, polygalacturonic acid, rhamnogalacturonan, xylan, and xyloglucan). Data were log transformed. The heatmap was drawn using the pheatmap package version 1.0.12 (https://cran.rstudio.com/web/packages/pheatmap/index.html) in R software version 4.1.2 (http://www.R-project.org/).

For qPCR analysis of the gene *NCU00810*, total RNA after induction of *N. crassa* in 0.5% arabinose or 0.5% xylan for 24 or 48 h was extracted using Trizol method with chloroform separation and isopropanol precipitation. Thirty to 100 mg of frozen harvested biomass was homogenized using a bead beater (three rounds of 30 s each at maximum speed) after adding 1 mL of Trizol (RNA-Solv® Reagent, Omega Bio-tek, Norcross, GA, USA), followed by DNAse I (NEB, Ipswich, MA, USA) treatment and column purification using the GeneJET RNA Purification Kit (ThermoFisher Scientific, Waltham, MA, USA), according to the manufacturer’s instructions. cDNA was synthesized with the reverse transcriptase FastGene® Scriptase Basic (Nippon Genetics, Tokyo, Japan). Real-time expression analyses were carried out using a MIC qPCR Cycler (Bio Molecular Systems, Upper Coomera, Australia) and qPCR SyGreen Mix No-ROX without ROX additive (PCR Biosystems, London, UK). Real-time (RT) PCR determinations were performed on at least three independent biological samples. RT-PCR experiments were carried out three times for each biological sample, with the threshold cycle (C_T_) determined in triplicate. The relative levels of transcription were calculated by using the 2^–∆∆CT^ method (Schmittgen and Livak 2008), and the standard error was computed from the average of the ∆C_T_ values for each biological sample. Gene expression was normalized to actin (*NCU04173*) transcript level. The PCR program consisted in a 2-min incubation at 95 °C, followed by 35 cycles of 5 s at 95 °C and 25 s at 60 °C, where the fluorescence signal was measured. The specificity of the PCR amplification procedure was checked with a heat-dissociation protocol (from 64.5 to 95 °C) after the final cycle of the PCR. Oligonucleotides used can be found in Supplemental Table S1.

### *N. crassa* growth assays

For the liquid growth assays, the strains were grown in 24 deep-well plates that were directly inoculated with 10^6^ conidia/mL in a volume of 3 mL medium. Cultures were grown in 1% lactose or 1% sucrose for 17 h and then, the mycelia were transferred to 2 mM lactose and incubated, unless otherwise indicated, at 25 °C, 200 rpm, and constant light. Incubation time is indicated for each assay. To determine the biomass (dry weight, DW) of the strains, the mycelial mass was dried for 16 h in aluminum pans at 105 °C and measured afterwards. All assays were done with 3–4 biological replicates for each strain per condition.

### Lactose uptake assays

Transport assays were performed essentially according to Benz et al. (2014). Briefly, *N. crassa* cultures were pre-grown from 9- to 10-day-old conidia with an initial OD_600_ = 0.03 for 16 h in 3 mL of Vogel’s salts plus 2% (w/v) sucrose using 24 deep-well plates. The mycelia were then washed three times in Vogel’s salts without carbon and transferred to the corresponding induction condition. After an additional 4 h, the mycelia were washed again as above and transferred into the uptake solution (3 mL of 0.5 × Vogel’s salts plus 100 μM lactose) for pre-equilibration. Three mycelia of the same genotype were combined into one well to increase the biomass at this stage. The 24 deep-well plates were incubated at 25 °C and 200 rpm with constant light. Several time points of the supernatants were taken. One hundred microliters of the supernatants was diluted into 900 μL of milli-Q water and the samples were cleared by centrifugation (5 min at 20,000 × g). Lactose concentration was then quantified by high-performance anion-exchange chromatography with pulsed amperometric detection (HPAEC-PAD) (Thermo Fisher Scientific Inc., Bannockburn, IL, USA). A sample size of 25 μL was injected onto a Dionex CarboPac PA20 column (Thermo Fisher Scientific Inc., Bannockburn, IL, USA) and eluted at 30 °C using an isocratic mobile phase of 100 mM NaOH at 1 mL/min over 15 min.

### Sugar uptake capacities

Assays for sugar uptake capacity studies were initiated as for the lactose uptake assays. In this case, only the wild-type (WT) strain was used. WT cells were pre-grown in Vogel’s salts with sucrose as the sole carbon source for 16 h and washed before induction. Induction of transporters’ expression was performed for 4 h in fifteen different induction conditions: no carbon, 2 mM d-galactose, 2 mM d-galacturonic acid, 2 mM d-glucose, 2 mM d-mannose, 2 mM d-xylose, 2 mM l-arabinose, 2 mM l-rhamnose, 0.1% cellulose, 0.1% glucomannan, 0.1% pectin, 0.1% xylan, 0.1% *Abies alba*, 0.1% *Miscanthus*, and 0.1% *Quercus robur*. After the induction phase, uptake rate of ten sugars—d-galactose, d-galacturonic acid, d-glucose, d-mannose, d-xylose, l-arabinose, l-rhamnose, cellobiose, lactose, and mannobiose—was measured, using the time points 0 and 15 min and three biological replicates for each measurement. The instrument method was modified depending on the sugar to measure.

In order to correlate transporter expression profiles with actual uptake, data from transporter expression profiles (Wu et al. 2020) and uptake rates per biomass using the same experimental setup were combined using the Hierarchical Clustering Explorer Version 3.5 software (Seo et al. 2006). The data were log transformed and normalized, and the Pearson correlation coefficient was used to measure similarity.

### Gene isolation, heterologous expression, and yeast growth assays

For growth assays in *S. cerevisiae*, the full-length cDNAs of *N. crassa cdt-1*, *cdt-2*, and *NCU00809* were amplified from cDNA using the corresponding primer pairs. Constructs were built by recombination-mediated plasmid construction in yeast (Oldenburg et al. 1997). The full-length cDNAs were cloned into the yeast cloning vector p426H7, which contains a fragment of the *HXT7* promoter, the *CYC1* terminator, and the *URA3* gene. The *LAC12* gene from *Kluyveromyces lactis* cloned into the same vector was used as positive control. All primers used for cloning of the constructs are listed in Supplemental Table S1.

Yeasts were transformed with the corresponding construct or the empty vector p426H7 and the *KlLAC4*-p425H7 construct, containing the β-galactosidase gene *LAC4* from *K. lactis* and the *LEU2* gene, using a lithium acetate–based method (Gietz and Woods 2002), and transformants were selected on synthetic complete (SC) medium with 2% maltose by autotrophy to uracil and leucine. For the heterologous gene expression assays, yeast mutant strain EBY.VW4000 (Wieczorke et al. 1999) and SC media without uracil and leucine supplemented with lactose or other sugars were used. Serial 1:5 dilutions of transformed cells (initial OD = 1) were spotted (5 µL) onto plates and incubated at 30 °C.

### Statistical analyses

Data shown in graphs represent the mean of at least three biological replicates and error bars correspond to the standard error. For data comparing two groups, for each parameter analyzed, each treatment was first subjected to the Shapiro–Wilk test for normality. If treatments had a normal distribution, a two-tailed Student *T*-test was performed. In case one (or both) of the treatments was not normally distributed, a Mann–Whitney *U* test was applied. Significance is indicated by asterisks (*p < 0.05; **p < 0.01; ***p < 0.001). For data comparing more than two groups, when showing a normal distribution, an ANOVA and Tuckey post hoc test were applied. Significant differences with *p* < 0.05 are indicated by different letters.

## Results

### Identification of putative sugar transporters in *N. crassa*

As a first step in the characterization of the *N. crassa* sugar transportome, a search for putative sugar transporter genes in the *N. crassa* genome was conducted. Forty-four genes that potentially encode sugar transporters were found (Supplemental Table S2). Only NCU00450 and NCU09321 belong to the glycoside-pentoside-hexuronide: cation symporter family (TC 2.A.2). The remaining forty-two candidate genes belong to several multigene families of the major facilitator superfamily. Concretely, putative sugar transporters NCU03468, NCU04310, NCU05394, and NCU5897 (FRT-1) belong to the fucose: H^+^ symporter family (TC 2.A.1.7) and the rest belong to the sugar porter family (TC 2.A.1.1). The length of the protein sequences ranges from 424 to 1050 amino acids, but most are between 520 and 674 amino acids in length. FungiDB identification numbers are listed in Supplemental Table S2. Out of the 44 putative sugar transporters, only 14 have been characterized to date (Supplemental Table S3), including three glucose transporters, five pentose transporters, one galacturonic acid transporter, one quinic acid transporter, three disaccharide transporters, and one glucose sensor. Interestingly, some transporters have a lower specificity and are able to transport different—albeit structurally related—substrates (Supplemental Table S3). All characterized *N. crassa* sugar transporters belong to the sugar porter family (TC 2.A.1.1) with the exception of FRT-1 (fucose-rhamnose transporter), which belongs to the fucose: H^+^ symporter family (TC 2.A.1.7; Wu et al. 2020) and is an ortholog of the l-rhamnose transporter previously identified in *A. niger*, RhtA (Sloothaak et al. 2016).

### Phylogenetic analysis of the *N. crassa* putative sugar transporters

To get a first insight into the possible functions of the uncharacterized sugar transporters in *N. crassa*, a phylogenetic analysis of fungal sugar transporters belonging to the major facilitator superfamily was carried out. For this purpose, we combined the 44 *N. crassa* transporters with 135 functionally characterized sugar transporters derived from an extensive literature search of more than eighty articles (Supplemental Table S4). The majority of genes were identified from *Ascomycota* fungi (36 species), most of them from *Saccharomycetes* (14 species) and *Sordariomycetes* (12 species), but also from seven *Basidiomycota* species.

**Fig. 1:**
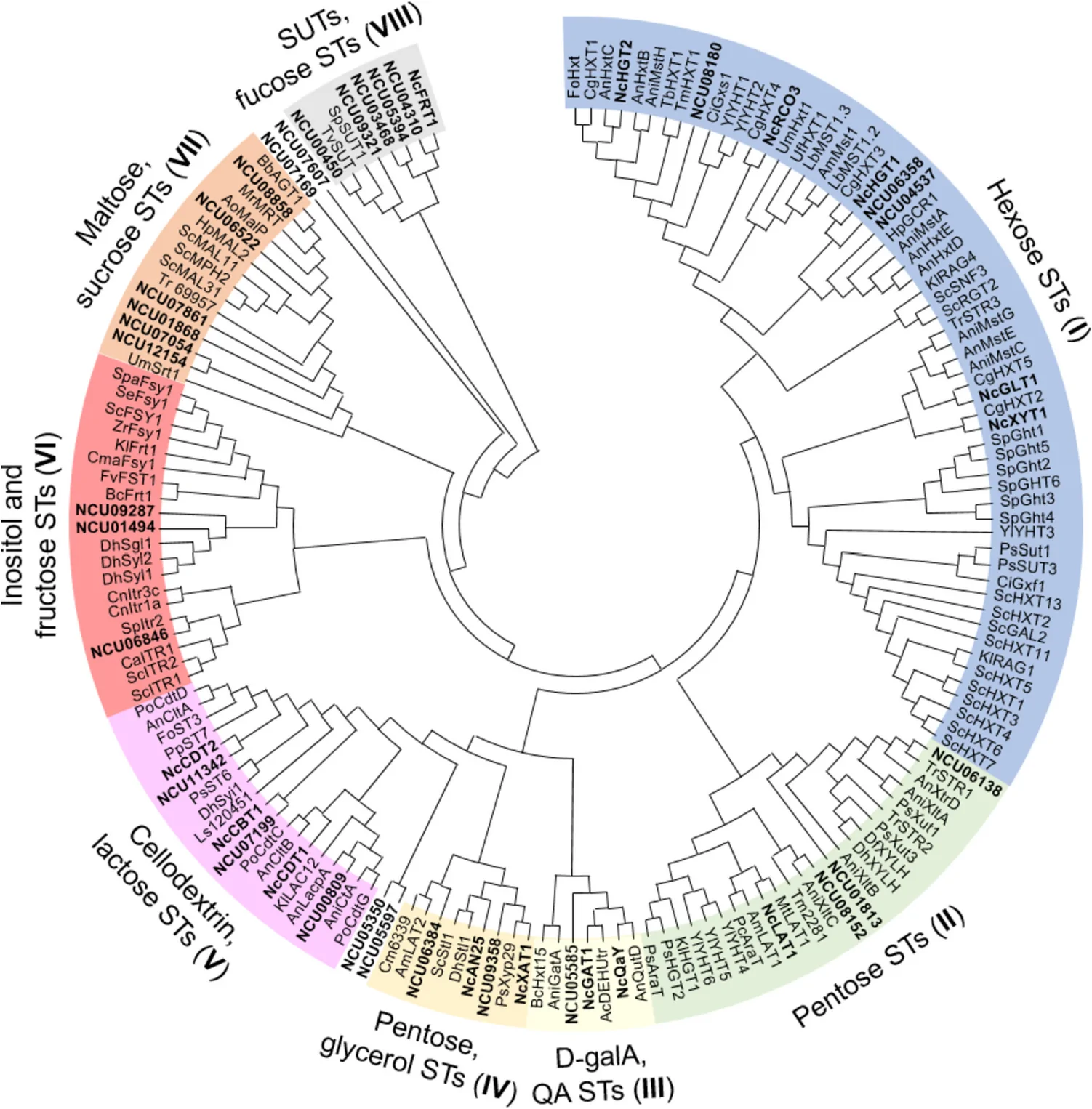
Phylogenetic relationships of the *Neurospora crassa* sugar transporter candidates with characterized sugar transporter sequences from different fungi. The unrooted neighbor-joining tree was created with MEGA11 (Tamura et al. 2021). *N. crassa* sugar transporter candidates are shown in bold. Roman numbers in brackets indicate phylogenetic clades. Organisms: Am *Amanita muscaria*, An *Aspergillus nidulans*, Ani *Aspergillus niger*, Ao *Aspergillus oryzae*, Ac *Asteromyces cruciatus*, Am *Ambrosiozyma monospora*, Bb *Beauveria bassiana*, Bc *Botrytis cinerea*, Ca *Candida albicans*, Ci *Candida intermedia*, Cma *Candida magnoliae JH110*, Cg *Colletotrichum graminicola*, Cm *Cordyceps militaris*, Cn *Cryptococcus neoformans*, Df *Debaryomyces fabryi*, Dh *Debaryomyces hansenii*, Fo *Fusarium oxysporium*, Fv *Fusarium verticillioides*, Hp *Ogataea polymorpha* (formerly *Hansenula polymorpha*), Kl *Kluyveromyces lactis*, Lb *Laccaria bicolor*, Ls *Lipomyces starkeyi*, Mr *Metarhizium robertsii*, Mt *Thermothelomyces thermophilus* (formerly *Myceliophthora thermophila*), Pc *Penicillium chrysogenum*, Po *Penicillium oxalicum*, Ps *Scheffersomyces stipitis* (formerly *Pichia stipitis*), Pp *Rhodonia placenta* (formerly *Postia placenta*), Sc *Saccharomyces cerevisiae*, Se *Saccharomyces eubayanus*, Spa *Saccharomyces pastorianus*, Sp *Schizosaccharomyces pombe*, Tr *Trichoderma reesei*, Tb *Tuber borchii*, Tv *Trichoderma virens*, Tm *Tuber melanosporum*, Uf *Uromyces fabae*, Um *Ustilago maydis*, Yl *Yarrowia lipolytica*, Zr *Zygosaccharomyces rouxii*. STs, sugar transporters

The phylogenetic analysis revealed eight clades similar to those described by Peng et al. (2018). Eight *N. crassa* sugar transporters clustered with typical hexose transporters, three with uronic acid and quinic acid transporters, eight with pentose and glycerol transporters, three with inositol and fructose transporters, six with SUT-type sucrose transporters and fucose transporters, and twelve with other disaccharide transporters (Fig. 1). Finally, four clustered outside of the identified clades and were orthologues of hypothetical (uncharacterized) proteins from other organisms. However, NCU05350 shows homology to fungal quinate permeases and NCU07607 to carboxylic acid transporters according to FungiDB (fungidb.org/).

### Expression profiles of putative *N. crassa* sugar transporter genes on different carbon sources

**Fig. 2:**
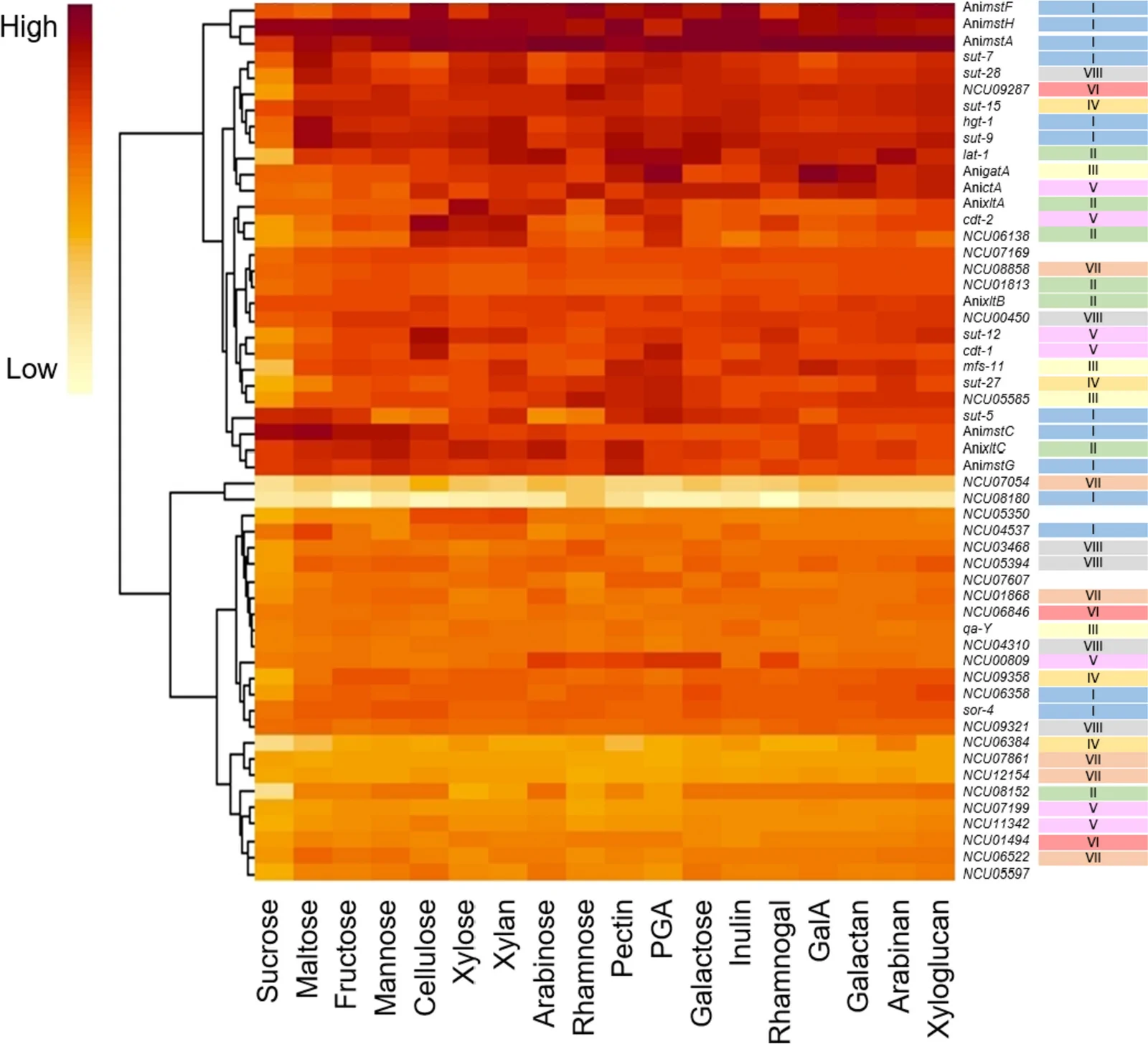
Expression analysis of *Neurospora crassa* sugar transporters under 18 different induction conditions. Gene expression data of the characterized *Aspergillus niger* sugar transporters from Peng et al. (2018) are included (Ani- prefix). Roman numbers on the right side of the figure indicate the phylogenetic clade of each sugar transporter according to Fig. 1

To further investigate the possible function of the putative sugar transporters, their available gene expression data in *N. crassa* under different carbon sources were examined (Wu et al. 2020). Gene expression profiles from growth on seven monosaccharides, two disaccharides, and nine complex sugars were chosen for the analysis, and expression profiles of ten characterized transporters from *A. niger* were included for comparison (Peng et al. 2018). The resulting heatmap revealed that most of the sugar transporter candidate genes are upregulated under specific sugar induction conditions, often—but not always (see below)—representing their own substrates or related polysaccharides (Fig. 2, Supplemental Table S1). For example, the d-galacturonic acid transporters GatA from *A. niger* and GAT-1 from *N. crassa* are transcriptionally induced by d-galacturonic acid and polygalacturonic acid (PGA), the CDTs (CDT-1 and CDT-2) by cellulose, and LAT-1 by arabinose and arabinan (Fig. 2). Further interesting expression patterns were found: Firstly, transporters from the same phylogenetic clade do not necessarily show similar expression profiles. Secondly, some transporter-encoding genes are constitutively expressed with high transcript numbers, as for example *sut-15* (AN25), while others are weakly expressed in all the induction conditions, such as *NCU07054* and *NCU08180*, suggesting a minor role under the tested conditions. Moreover, most genes (70%) are clearly downregulated in the presence of sucrose, indicating a repression by CCR. Some exceptions include the low-affinity glucose transporter–encoding genes *mstC* from *A. niger* and *sut-5* (GLT-1) from *N. crassa*. Glucose transporter–encoding genes, such as *A. niger mstC* and *N. crassa hgt-1*, *sut-9* (HGT-2) and *sut-5* (GLT-1), are upregulated under maltose induction. Remarkably, some of the characterized transporter-encoding genes do not show peak expression in presence of their substrates. For example, *sut-28* (FRT-1) is not induced under rhamnose induction conditions, or *sut-7* (XYT-1) and *sut-15* (AN25) in the presence of xylose, indicating that the observed induction conditions are not always a clue to the substrate of a particular transporter.

### Characterization of sugar transport capacities of *N. crassa*

**Fig. 3:**
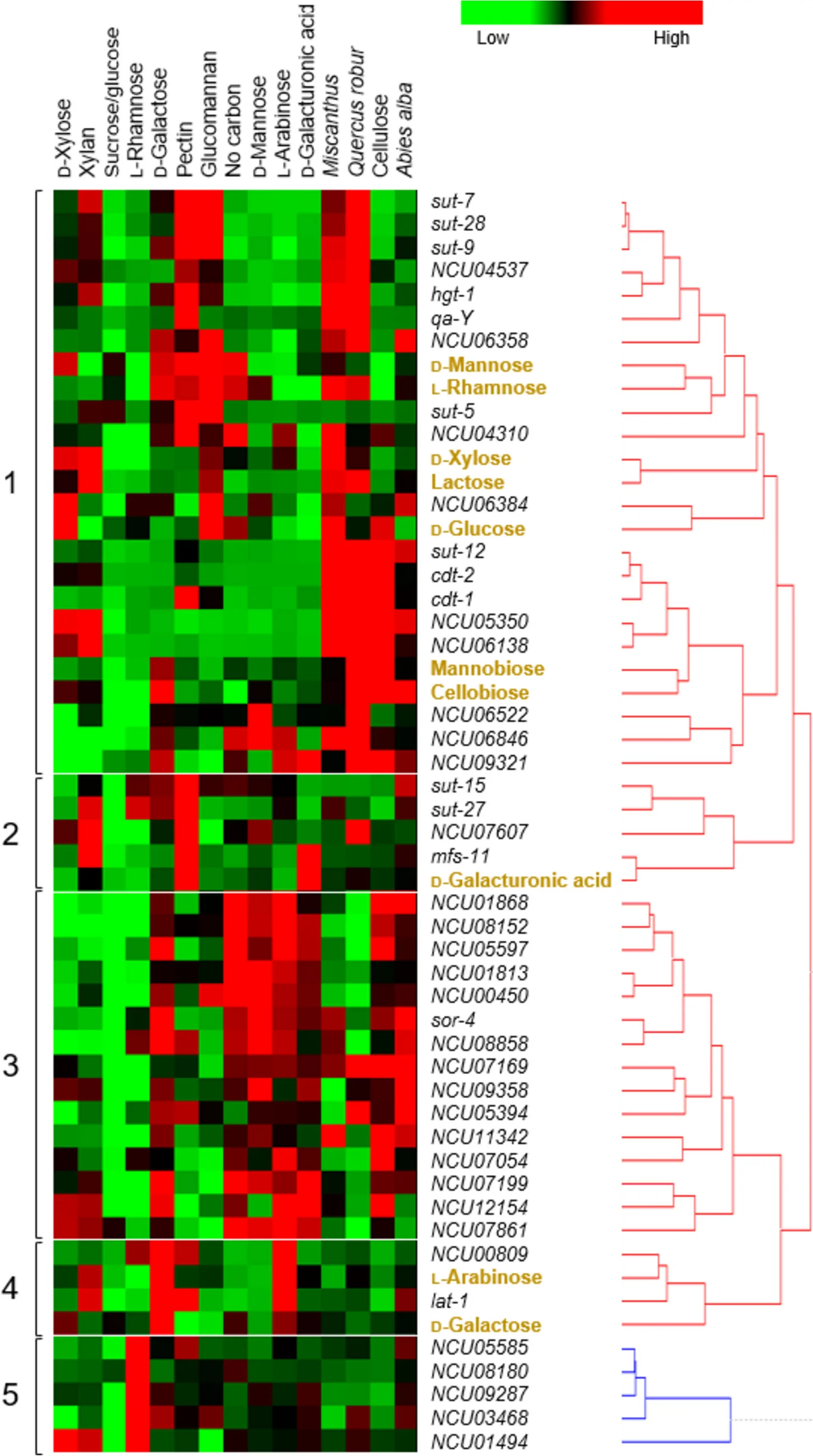
Clustering of expression profiles of *Neurospora crassa* sugar transporter-encoding genes correlated with uptake rates. Gene expression data under fifteen different induction conditions (horizontal axis) and uptake rate values after 15 min of ten different sugars in the *N. crassa* WT strain after 4-h induction under the same induction conditions were used. Numbers 1–5 indicate different clusters. Sugars are shown in gold color

To achieve a better predictability of transporter function than possible by phylogeny and transcriptional analysis, we wondered whether gene expression could be correlated with sugar uptake rates. We therefore decided to combine transcriptomic data with actively measured sugar uptake capacities to capture the dynamics of the “sugar transportome.” For this purpose, the uptake rates of ten reference sugars—seven monosaccharides and three disaccharides—were recorded for the *N. crassa* WT strain after induction under fifteen conditions, including some complex sugars, in order to have a larger dataset (Supplemental Fig. S1 and S2). Subsequently, these sugar uptake rates were combined with the expression data of the *N. crassa* sugar transporter candidate genes. We observed that sugar uptake rates could indeed be correlated with gene expression (Fig. 3). For example, the l-arabinose uptake rates clustered along with the expression profile of the l-arabinose transporter gene *lat-1* within cluster 4. The same was observed for the uptake rates of d-galacturonic acid and the expression of the galacturonic acid transporter GAT-1 (encoded by *mfs-11*, cluster 2). Moreover, *cdt-1* and *cdt-2* were in the same cluster (1) and in close proximity to the uptake rates of their substrates cellobiose and mannobiose, which corroborated that the method works in many cases.

In cases in which several transporters are involved in the uptake of a certain substrate however, the uptake rate would be the sum of the individual rates and consequently, the correlations would appear to be less direct in these cases. Nevertheless, the activity profiles and expression of the involved transporter genes tended to be in the same cluster. This is the case, for example, for the glucose transporters HGT-1, HGT-2, and GLT-1, encoded by *hgt-1*, *sut-9*, and *sut-5*, respectively (cluster 1) (Xie et al. 2004; Du et al. 2010; Li et al. 2014; Wang et al. 2017). In the case of d-xylose, many transporters are involved; known are, for example, AN25, XAT-1 (which can also transport l-arabinose in yeast), and XYT-1 (Du et al. 2010; Li et al. 2014). The clustering distance to the substrate of different permeases transporting the same substrate could indicate that their physiological contribution is not equal.

In the case of l-rhamnose, its major physiological transporter in *N. crassa* (FRT-1, encoded by *sut-28*) was found not to be induced by its substrate (see above) and also clustered not very close to it in this analysis, suggesting that post-transcriptional or post-translational regulations of FRT-1 may be more important than transcriptional regulation.

d-Mannose- and d-galactose-specific transporters are unknown in *N. crassa*. d-Galactose was found in cluster 4 near *lat*-1 and *NCU00809*. LAT-1 had been previously found to allow d-galactose uptake in a heterologous system (Li et al. 2015) and may thus non-specifically carry out the uptake of D-galactose in *N. crassa*. For d-mannose, putative monosaccharide transporters from cluster 1 (like XYT-1, FRT-1, HGT-2, NCU04537, HGT-1, QaY, NCU06358, GLT-1, NCU06384, and NCU06138) could be potential candidates, but likely also only by contributing with unspecific side activities.

### Lactose uptake assays of transporter candidates

**Fig. 4:**
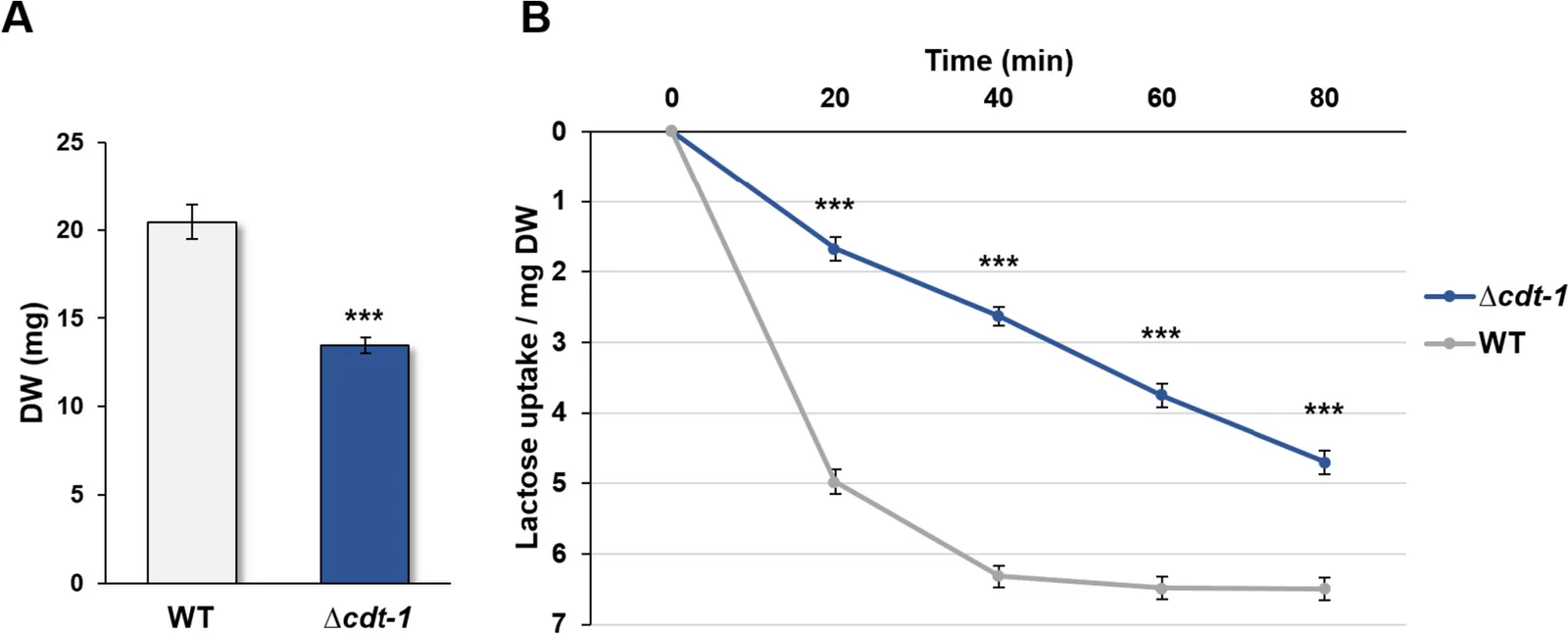
Growth on lactose and lactose transport analysis of the *Neurospora crassa* ∆*cdt-1* mutant. **A** Growth of the ∆*cdt-1* mutant on MM + 1% lactose as sole carbon source for 7 days. **B** Lactose transport after induction in cellulose. Asterisks indicate statistical significance compared to the WT strain (*n* = 3), ***p < 0.001. DW, dry weight

Following the previous global analyses of the sugar transportome of *N. crassa*, we were interested in elucidating the transport system of the important dairy by-product lactose, which is so far unknown in *N. crassa*. Based on the combined expression and transport profiling (Fig. 3), lactose uptake was located in cluster 1, together with several disaccharide transporters, including the CDTs. Since Liu et al. (2016) observed lactose uptake by CDT-1 in a heterologous system, the growth of the *N. crassa* ∆*cdt-1* mutant on lactose as sole C-source was tested first. The growth was found to be reduced to about 65% of WT levels (Fig. 4A and Supplemental Fig. S3A), suggesting that other transporters might be involved in lactose uptake as well. Similarly, in lactose uptake assays after cellulose induction of the WT and the single ∆*cdt-1* mutant, we found that lactose transport decreased in the ∆*cdt-1* mutant, but was not abolished (Fig. 4B), corroborating the notion that more proteins are involved in lactose uptake.

In the search for new lactose transporter candidates, we first focused on phylogenetic and gene expression analyses. Phylogenetic analysis showed that only two *N. crassa* proteins, CDT-1 and NCU00809, cluster together with the characterized fungal lactose transporters Lac12 from *K. lactis* and LacpA from *A. nidulans* (Fig. 1). In terms of expression, lactose transporters in *T. reesei* are induced by cellulose (Havukainen et al. 2021), and in *A. nidulans*, the monosaccharides d-galactose and l-arabinose have been found to induce the expression of the *bgaD-lacpA* gene couple (encoding an intracellular β-galactosidase and a lactose permease) (Fekete et al. 2012; Orosz et al. 2014). *NCU00809* gene expression is highly induced by these two monosaccharides (Fig. 2) and its expression strongly co-regulated with their uptake (Fig. 3). Moreover, *NCU00809* is adjacent in the genome to *NCU00810*, encoding a protein with a predicted intracellular location and 52% identity with the β-galactosidase BgaD from *A. nidulans* (Orosz et al. 2014). The existence of gene clusters with disaccharide transporters and hydrolases has been observed not only for lactose permeases but also for other disaccharide transporters and in many other fungal species, probably due to an evolutionary pressure for proximity of these functionally related genes (Donzella et al. 2023).

**Fig. 5:**
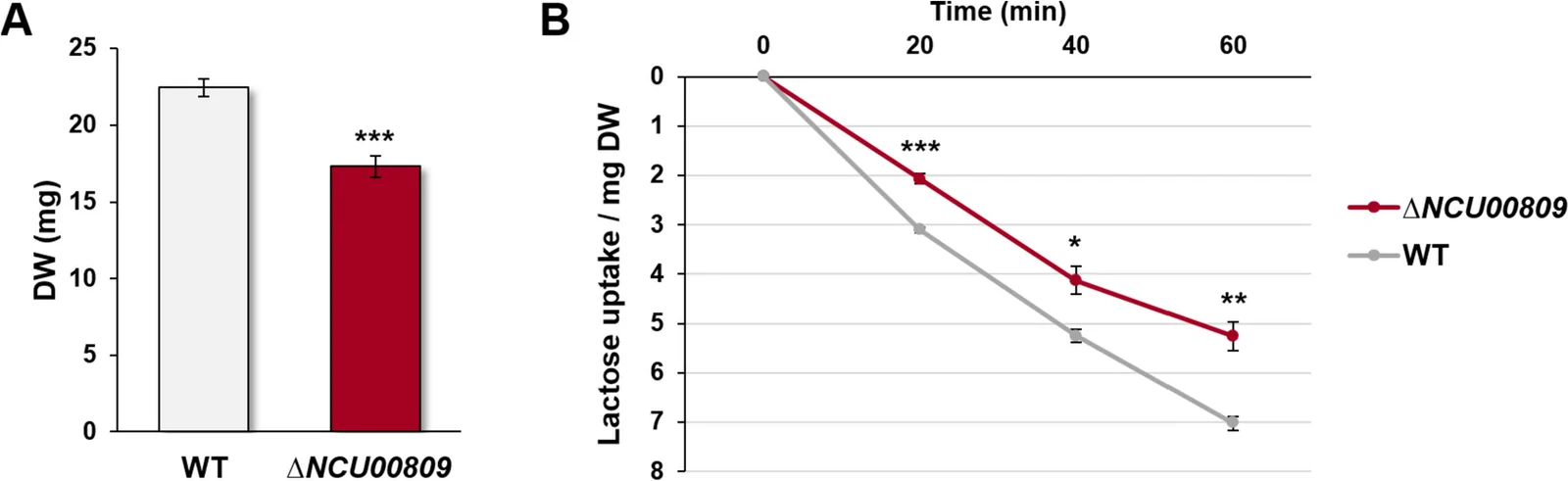
Growth on lactose and lactose transport analysis of the *Neurospora crassa* ∆*NCU00809* mutant strain. **A** Growth of the ∆*NCU00809* mutant on MM + 1% lactose as sole carbon source for 7 days. **B** Lactose transport after induction in arabinose. Asterisks indicate statistical significance compared to the WT strain (*n* = 3), *p < 0.05; **p < 0.01; ***p < 0.001. DW, dry weight

When tested with lactose as the sole carbon source, the Δ*NCU00809* mutant exhibited a clear growth defect (Fig. 5A), reduced to approx. 75% of WT levels, which we confirmed not to be due to lower expression of the neighboring β-glucosidase-encoding gene *NCU00810* in this mutant (Supplemental Fig. S4). Also, the lactose uptake was found to be reduced compared to the WT strain under optimal conditions for *NCU00809* gene induction (l-arabinose) (Fig. 5B), albeit not as strong as seen for the ∆*cdt-1* mutant (Fig. 4B). When considering furthermore that the fastest transport of lactose was achieved after induction with xylose and particularly xylan (Fig. 3 and Supplemental Fig. S2C) and that neither *NCU00809* nor *cdt-1* are induced by xylan (Fig. 2), it was clear that another important player in lactose transport that is induced under that condition should be present.

Out of the transporters belonging to the cellodextrin- and lactose-transporter clade V (Fig. 1), CBT-1 and CDT-2 were identified as additional candidates. However, despite the fact that the cellobionic acid transporter CBT-1 clustered near lactose (Fig. 3), it has very low homology with characterized lactose transporters, with only 26 and 24% identity with the LacpA transporter from *A. nidulans* and the Lac12 transporter from *K. lactis*, respectively. Moreover, the ∆*cbt-1* mutant did not show significantly different growth compared to the WT strain when grown on lactose as the sole carbon source (Supplemental Fig. S5). For these reasons, further experiments were performed with the Δ*cdt-2* deletion strain, together with the Δ*cdt-1* and Δ*NCU00809* strains for comparison.

### Further analysis of the more promising lactose transporter candidates

**Fig. 6:**
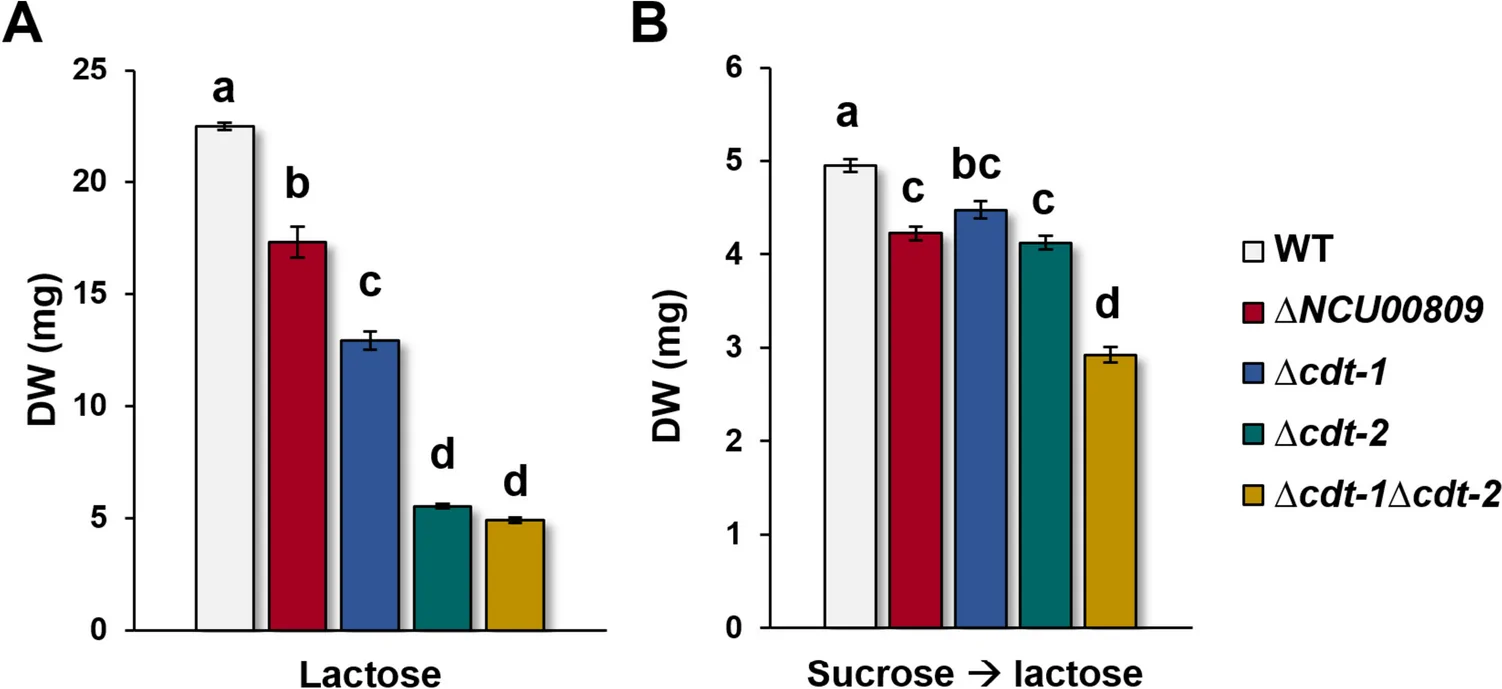
Growth assays of different *Neurospora crassa* mutant strains in lactose media. **A** Spores were inoculated into MM + 1% lactose as sole carbon source and grown for 7 days. **B** Spores were initially inoculated into MM + 2% sucrose for 17 h to build up a comparable amount of biomass and then switched to MM + 2 mM lactose as sole carbon source for another 7 days. The final biomass differed considerably on the various media due to the different growth speeds on sucrose and lactose, being much higher on sucrose, where after only 2 days, the cultures begin to sporulate, while more time is needed for growth on lactose. Different letters indicate statistically significant differences (calculated as explained in the “Material and methods” section). DW, dry weight

All three mutant strains grew significantly worse than the WT, both when conidia were inoculated directly into medium with lactose as sole carbon source (Fig. 6A) and when mycelium was switched to lactose medium after 17 h of pre-incubation in sucrose (Fig. 6B and Supplemental Fig. S3A). Differences in growth were less severe in the latter case due to the pre-growth in sucrose, where all mutants grew similarly (cf. Supplemental Fig. S3A), but were nevertheless clearly visible. Interestingly, the ∆*cdt-2* mutant strain and the ∆*cdt-1*∆*cdt-2* double mutant strain showed similar and the lowest growth on medium with lactose as the sole carbon source. These results indicated strongly that CDT-2 is a key player in lactose transport and/or metabolism in *N. crassa*.

**Fig. 7:**
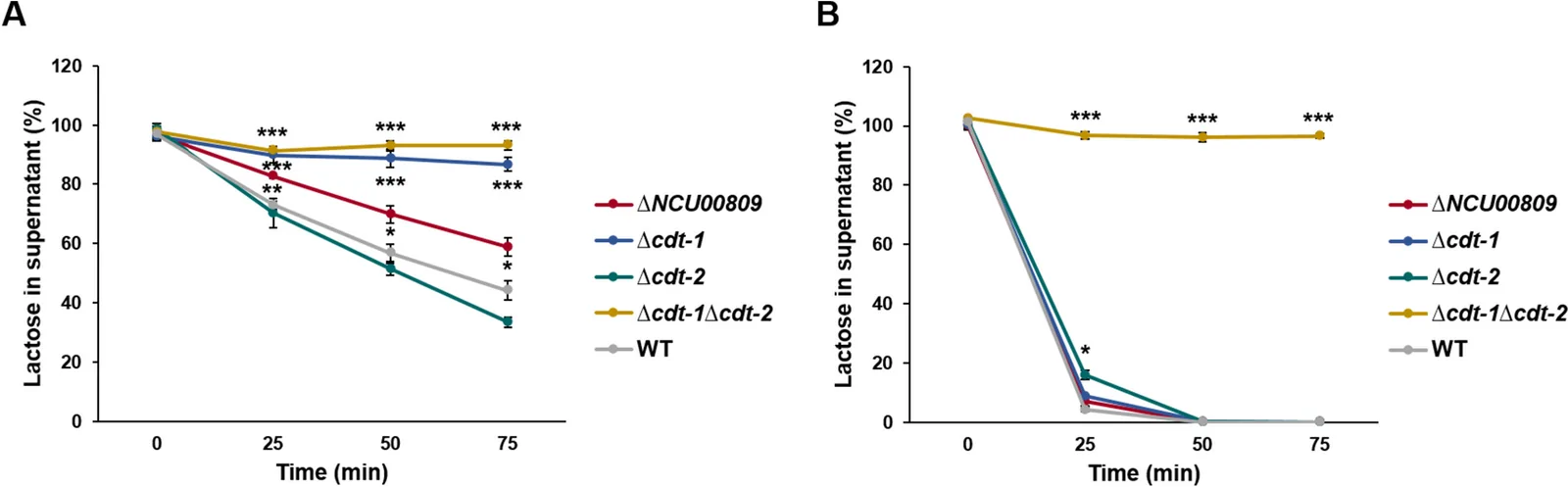
Lactose uptake of different *Neurospora crassa* mutant strains in selected genes with homology to lactose transporters. Values of lactose remaining in supernatant (%) under arabinose (**A**) and xylan (**B**) induction conditions are shown. Asterisks indicate statistical significance compared to the WT strain (*n* = 3), *p < 0.05; **p < 0.01; ***p < 0.001

To analyze the involvement of CDT-1, CDT-2, and NCU00809 in lactose transport in *N. crassa*, we then performed comparative uptake assays of this disaccharide with the candidate mutant strains. To this end, lactose concentrations in culture supernatants were measured by HPAEC-PAD over time in two different induction conditions, favorable and unfavorable for lactose transport (Fig. 7). In the presence of l-arabinose, taken as unfavorable condition (Fig. 3 and Supplemental Fig. S4), only about 55% of the lactose in the supernatant was taken up by the WT strain after 75 min. As previously observed, the ∆*NCU00809* mutant showed a significantly reduced lactose transport compared to the WT strain in this condition. Nevertheless, the greatest reduction was observed for the ∆*cdt-1* single and ∆*cdt-1*∆*cdt-2* double mutant, with almost no uptake observed in the double mutant. This also occurred after induction in 2 mM lactose (Supplemental Fig. S6). On the other hand, after induction with xylan, taken as the most favorable condition for lactose transport, almost 100% of the lactose in the supernatant was taken up by the WT strain already after 25 min. In this case, transport was significantly reduced only in the single ∆*cdt-2* mutant strain and almost completely abolished in the ∆*cdt-1*∆*cdt-2* double mutant. After optimal induction for both transporters (with cellulose), both have a similar importance in lactose uptake (Supplemental Fig. S7). Taken together, these results indicate that the major share of lactose transport in *N. crassa* is carried out by CDT-1 and CDT-2 transporters, while NCU00809 has a minor role in it—particularly in conditions with little *cdt-1*/*cdt-2* expression. This highlights the importance to carefully consider the induction conditions of the candidate genes. Moreover, these insights further substantiate the low specificity of these sugar transporters, as these have previously been observed to accept not only cellodextrins but also xylodextrins and mannodextrins as substrates (Ha et al. 2011; Cai et al. 2014; Hassan et al. 2019).

**Fig. 8:**
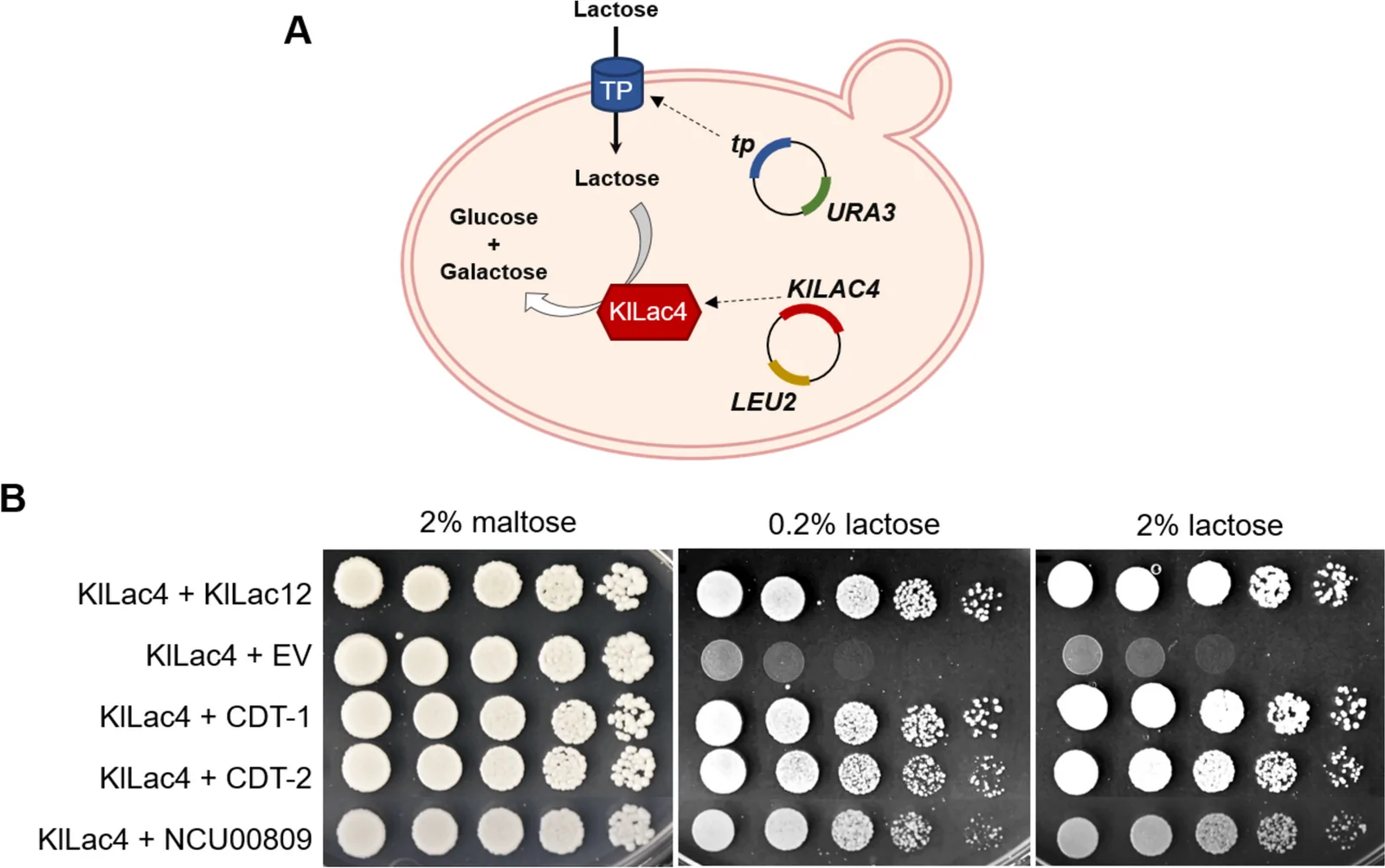
Analysis of the lactose transport ability of three *Neurospora crassa* transporter candidates in the yeast *Saccharomyces cerevisiae*. The yeast strain EBY.VW4000 was transformed with the β-galactosidase *LAC4* gene from *Kluyveromyces lactis* and with an empty vector (p426H7) or with the positive control *KlLAC12* or with the *N. crassa* transporter genes *cdt-1*, *cdt-2*, or *NCU00809*, all cloned into p426H7. **A** Scheme of functioning of the system in yeast. When a transporter (TP), cloned in a vector with *URA3* as autotrophy marker, is able to transport lactose, this sugar will be internalized and hydrolyzed to glucose and galactose by Lac4, whose encoded gene was cloned in a vector with *LEU2* as autotrophy marker. *S. cerevisiae* is then able to metabolize the two monosaccharides and to grow in a medium with lactose as the sole carbon source. **B** Strains were spotted on SC medium without uracil and leucine supplemented with 2% maltose (growth control) or with 0.2% or 2% lactose and incubated at 30 °C for 3 days

Finally, to corroborate that the three transporters CDT-1, CDT-2, and NCU00809 are indeed able to transport lactose, heterologous expression assays were performed in *S. cerevisiae*. Since yeast cannot metabolize lactose natively, the β-galactosidase Lac4 from *K. lactis*, which can perform hydrolysis of lactose to glucose and galactose, was used (Varela et al. 2019b; Fig. 8A). Similar to the lactose transporter Lac12 from *K. lactis*, which was used as positive control, the three lactose transporter candidates from *N. crassa* enabled the yeast mutant strain EBY.VW4000 to grow with lactose as the sole carbon source (Fig. 8B), demonstrating that they are plasma membrane permeases that can transport lactose. In addition, NCU00809 was found to display transport activity for d-galactose (Supplemental Fig. S8), in agreement with previous clustering results (Fig. 3). CDT-1 again proved to be a rather promiscuous transporter, capable of transporting also sucrose and (at high concentrations) the monosaccharides mannose, glucose, and galactose (Supplemental Fig. S8).

### Extension of the method to non-MFS transporters

**Fig. 9:**
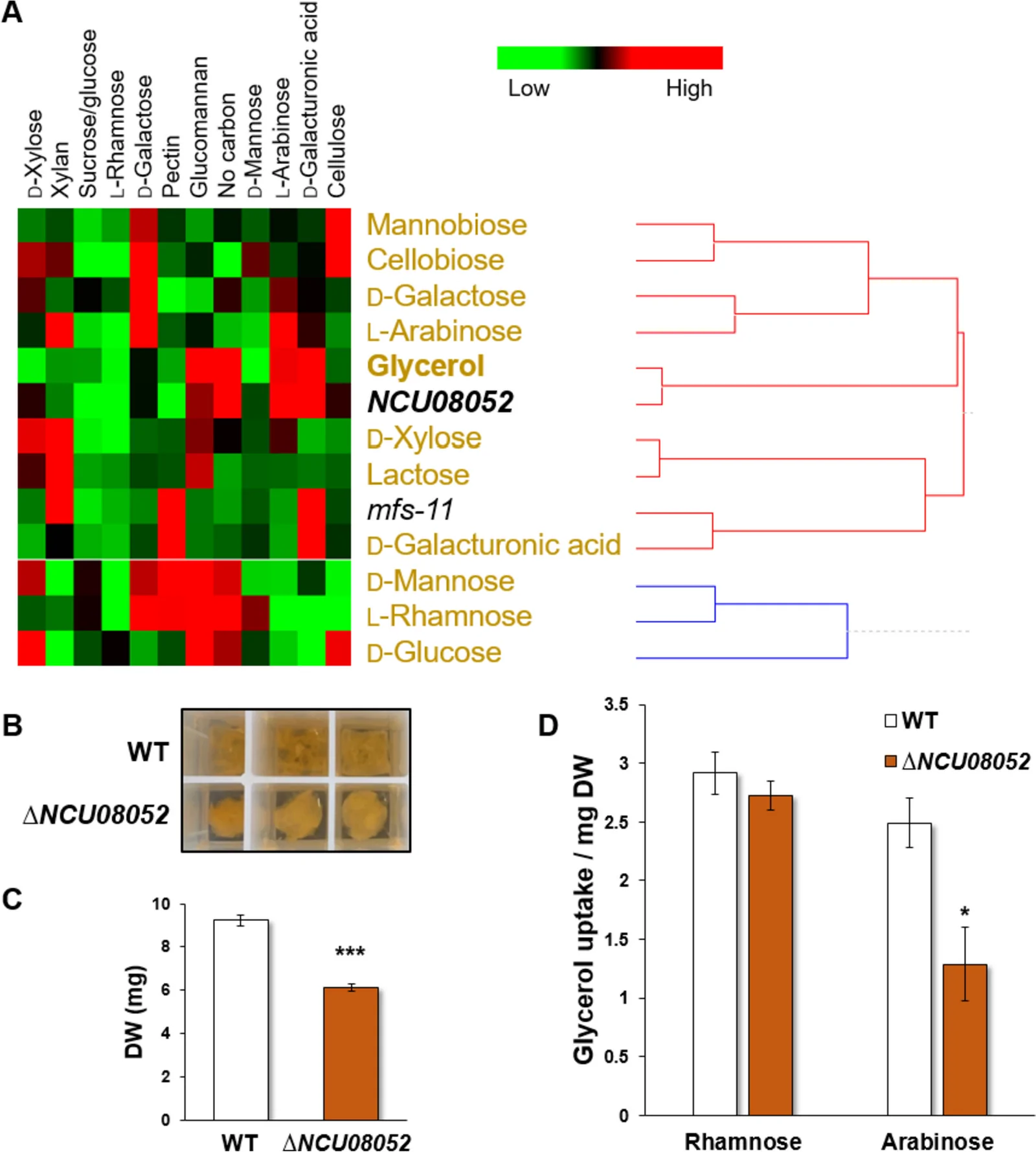
Analysis of the putative aquaporin NCU08052 from *Neurospora crassa*.** A** Clustering of gene expression profiles of the *N. crassa* aquaporin-encoding gene (*NCU08052*) correlated with uptake rates. Gene expression data under twelve different induction conditions (horizontal axis) and uptake rate values of eleven different sugars in the *N. crassa* WT strain after induction under the same induction conditions were used. Sugars are shown in gold color. *NCU08052* is indicated in bold. **B**, **C** Growth of the ∆*NCU08052* mutant on MM + 0.5% glycerol as sole carbon source for 2 days. **D** Glycerol uptake per milligram biomass and 20 min after induction in rhamnose or arabinose, taken as non-optimal and optimal induction conditions, respectively. Asterisks indicate statistical significance compared to the WT strain (*n* = 3), *p < 0.05; ***p < 0.001. DW, dry weight

To test whether the method can also be applied to non-MFS transporters, we decided to investigate a putative transporter of the Major Intrinsic Protein (MIP) family comprising aquaporin proteins, water channels that can also transport small uncharged polyols including sugar metabolites (Pettersson et al. 2005; Wei et al. 2013). NCU08052 is the only protein of the family encoded in the *N. crassa* genome, differing from other yeasts and filamentous fungi that may possess up to five aquaporins. Fungal aquaporins are divided into Fps1-like aquaglyceroporins, Yfl054c-like aquaglyceroporins (with a very long N-terminal extension including a conserved stretch), and a third group of proteins, which do not fall into any of these categories. NCU08052 belongs to the third category (Pettersson et al. 2005). Since several aquaporins have been shown to transport glycerol, we performed an identical uptake profile of this substrate in *N. crassa* WT induced with diverse carbohydrates as performed above (Fig. 3) and combined this, along with the respective profiles of all other tested sugars, with the expression profiles of *NCU08052* and *mfs-11* (GAT-1) as transporter control (Fig. 9A). We observed that *NCU08052* clustered directly adjacent to glycerol, suggesting that the protein could indeed transport this substrate. Growth assays with glycerol as the sole carbon source and uptake assays under an optimal induction condition for the gene—arabinose—confirmed this hypothesis by showing a clear growth defect (Fig. 9B, C and Supplemental Fig. S3B) and a significantly reduced uptake rate in the respective deletion mutant (Fig. 9D), suggesting that the method can be extended to transporter proteins belonging to other superfamilies.

## Discussion

In-depth studies of sugar transport are of great importance for potential biotechnological applications of fungi, as some yeasts with industrial interest, such as *S. cerevisiae*, have evolved to use only a limited number of sugars, resulting in significant losses of entire transporter families. However, there are many limitations to deorphanizing transporters or finding new substrates for known sugar transporters that need to be taken into account, including the low specificity of some of them and the redundancy among transporters in the sense that several can transport the same substrate. Furthermore, some sugar transporters are involved in intracellular transport and thus do not have a plasma membrane localization, and some may have a transceptor rather than a transporter function.

Previously, approaches used to identify substrates for a particular transporter included in silico analyses of transport proteins to identify substrate-binding motifs or transformation of the *S. cerevisiae* monosaccharide transporter null mutant EBY.VW4000 (Wieczorke et al. 1999; Li et al. 2015; Nogueira et al. 2020; Xiao et al. 2022). In the present study, a new approach was used to search for transporter-substrate pairs in *N. crassa* after using classical phylogenetic and expression analyses. The novel expression-uptake rate correlation method presented in this work can give new clues for this purpose and has the advantage of being a straightforward strategy using the study organism itself and data from uncomplicated laboratory experiments with WT strains.

Forty-four sequences putatively encoding sugar transporters were found in the genome of the filamentous fungus *N. crassa*, forty-two of them belonging to the MFS. This number differs from other *Ascomycota* fungi, being lower than in *A. niger*, which has a total of 86 identified sugar transporters, including eighteen with homology to disaccharide transporters, eight to polyol transporters, and sixty whose substrate is a monosaccharide (Peng et al. 2018). Peng et al. (2018) performed an extensive in silico study of sugar transporters by phylogenetic and comparative transcriptomic analyses and found that the transporter genes are regulated by different transcription factors, pointing to the nutritional adaptability of *A. niger*. By searching for the sugar transporter domain PF00083 (El-Gebali et al. 2019), Nogueira et al. (2020) found that *T. reesei* contains 64 predicted MFS transporters in its genome and *A. nidulans* 112. Surprisingly, 127 putative sugar transporters were found in the genome of *A. oryzae* (Lv et al. 2020). On the other hand, the model yeast *S. cerevisiae* has only 25 sugar transporters in its genome, most of them (17) hexose transporters, two sensors, only three disaccharide transporters, and three polyol transporters (Barbi et al. 2021; Donzella et al. 2023). The basidiomycete *Ustilago maydis*, a pathogen of maize, also possesses a reduced number of sugar transporters (20), some of them capable of transporting pentoses and uronic acids, in contrast to *S. cerevisiae* (Wahl et al. 2010).

A robust phylogenetic analysis including characterized sugar transporters from other fungi allowed us to classify *N. crassa* transporters into distinct phylogenetic groups. Overall, one-third of the *N. crassa* sugar transporters are putative disaccharide transporters, compared to the limited disaccharide assimilating capacity of *Saccharomycotina* yeasts (Donzella et al. 2023), which might indicate that disaccharide transport is of great importance to *N. crassa*. Furthermore, being able to utilize a wide diversity of disaccharide or trisaccharide units may increase efficiency in utilizing complex carbon sources from agricultural and human food waste. So far, only three such transporters have been characterized in *N. crassa*: CBT-1, CDT-1, and CDT-2 (Galazka et al. 2010; Li et al. 2015), warranting additional efforts to deorphanize missing oligosaccharide transporters.

Our transcriptional analyses allowed us to observe that not all transporters are induced by their known substrates. For this reason, and following the observation that different inducers can lead to distinct uptake profiles for the analyzed sugars, we combined the transcriptomic data with uptake rates of different sugars in order to have more information for the analysis of the *N. crassa* sugar transportome. While the method was shown to work well in several cases, e.g., LAT-1 and GAT-1, different cellular processes may affect the data, as, for example, in the case of the rhamnose/fucose transporter FRT-1, which did not cluster close to rhamnose. Several studies have shown that post-transcriptional and post-translational regulation occurs in *N. crassa* (Borkovich et al. 2004; Xiong et al. 2014; Du et al. 2015; Horta et al. 2019; Wang et al. 2021). For example, Xiong et al. (2014) observed post-transcriptional regulation in response to cellulose, including differential phosphorylation in the cellobionic acid transporter CBT-1. The fact that some transporters might be activated or inactivated by these transcription-independent mechanisms may thus influence the localization of their substrates in the clustering. The many instances in which we were able to observe a close clustering nevertheless suggest gene expression to be a major way of regulation.

The two monosaccharides mannose and galactose do not seem to be preferred by *N. crassa* and, for this reason, could be transported by general monosaccharide transporters rather than specific transporters. Several candidates emerged from our study, but so far, only LAT-1 had been found to be able to transport galactose non-specifically (Li et al. 2015). Our results showed for the first time that also NCU00809 can transport galactose, an activity that had also been suggested by our correlation of gene expression and transport activity (Fig. 3, cluster 4). With respect to the monosaccharide mannose, Barbi et al. (2021) observed a close association between glucose and mannose transport and found that out of the 49 published glucose transporters, only five could not transport mannose, including the l-arabinose transporter LAT-1 from *N. crassa*. This observation suggests that some of the glucose transporters from *N. crassa* may indeed also transport mannose. Moreover, the same authors observed that out of the 52 characterized fungal monosaccharide transporters that were tested for uptake of the hexoses d-fructose, d-galactose, d-glucose, and d-mannose, only one was specific for mannose, namely, YHT6 from *Yarrowia lipolytica* (Lazar et al. 2017), and only one for galactose, Hxt14 from *S. cerevisiae* (Wieczorke et al. 1999). The results of heterologous expression analysis performed in our work indicated that CDT-1 is able to transport mannose in a non-specific manner. Taken together, these observations support our hypothesis that mostly non-specific transporters contribute greatly to galactose and mannose uptake in *N. crassa*. However, transport specificity of other transporter candidates found will require further functional characterization.

To test the usefulness of our method, we then decided to focus on the study of uptake of lactose. Lactose is a by-product of the dairy industry, which is utilized for the induction of cellulase enzyme expression in some fungi (e.g., *T. reesei*; Warzywoda et al. 1983; Seiboth et al. 2002; Ivanova et al. 2013), but could also serve as a substrate for fungal biotransformations into products for the bioeconomy. In addition to lactic bacteria and enterobacteria, some fungi such as *A. niger*, *A. oryzae*, and *T. reesei* are able to metabolize lactose (Zou and Chang 2022). Nevertheless, the yeast *S. cerevisiae*, widely used in industrial fermentation processes, cannot metabolize lactose natively. This is because it does not have a β-galactosidase gene and lacks mechanisms to transport lactose across the plasma membrane. Several metabolic engineering approaches have been used to construct lactose-metabolizing *S. cerevisiae* strains, including genes from other fungi able to assimilate lactose. The yeast *K. lactis* can natively grow on lactose as a sole carbon source, because it possesses a β-galactosidase (*LAC4*) and the permease *LAC12* in its genome, and as a result, the construction of *S. cerevisiae* strains capable of growing on lactose was possible (Sreekrishna and Dickson 1985). Liu et al. (2016) took advantage of *N. crassa* in order to engineer a yeast strain able to ferment lactose, by expression of *cdt-1* and *gh1-1*, encoding a β-glucosidase that also acts as a β-galactosidase. After integration of the gene *ldhA* from *Rhizopus oryzae*, encoding a lactate dehydrogenase, the engineered strain could produce lactic acid not only from purified lactose but also from dairy by-products such as whey and store-bought milk (Turner et al. 2017). However, more knowledge is needed to optimize systems like these for higher productivity.

In nature, *N. crassa* normally lives in lactose-free environments and, for this reason, its lactose uptake and metabolism are low and its genome does not appear to encode for any specific lactose transporters. However, under laboratory conditions where lactose is the only carbon source, *N. crassa* is able to take it up, metabolize it, and grow (Lester et al. 1962). Thanks to the combination of gene expression and sugar transportome data, we showed that CDT-2 is equally important as CDT-1. This dual function to transport both cellobiose and lactose was also found for Lac12 from *K. lactis* and *K. marxianus* and CRT1 from *T. reesei* (Sadie et al. 2011; Varela et al. 2019b; Havukainen et al. 2020). The low specificity of CDT-1 and CDT-2 was already apparent from other studies, demonstrating transport capacity for mannobiose and xylobiose, respectively (Galazka et al. 2010; Cai et al. 2014; Hassan et al. 2019). In addition to the CDTs, NCU00809 also plays a role in lactose transport and metabolism, although reduced uptake in the ∆*NCU00809* mutant strain was only observed under optimal induction conditions, in which the CDTs are not expressed. Nevertheless, these findings provide new targets for the generation of genetically modified strains to optimize the reuse of dairy industry by-products in order to create economic value and reduce their environmental impact.

In conclusion, the strategy of combining gene expression and sugar transportome data presented in this study can support the identification of transporter candidates for a given sugar and, together with phylogenetic analysis, adds crucial data to this puzzle. However, it should be noted that transcriptional regulation is not the only regulation that can occur. As shown, this approach cannot only be used for MFS transporters but also for other transporter superfamilies and could be extended to other organisms. This will open new possibilities for the identification of transporter(s) for a given substrate in *N. crassa* as well as in other biotechnologically relevant filamentous fungi. Taking into account the limitations mentioned above, the addition of proteomic data in a next step may help to further complement our approach to fully elucidate the fungal sugar transportomes.

## Supplementary Information

Below is the link to the electronic supplementary material.

Supplementary file1 (PDF 1250 KB)

Supplementary file2 (XLSX 29 KB)

## Data Availability

The datasets generated during and/or analyzed during the current study are available from the corresponding author on reasonable request.
