# Supplementary material for: Correlating sugar transporter expression and activities to identify transporters for an orphan sugar substrate
Source: Appl Microbiol Biotechnol. 2024 Jan 8;108(1):83. doi: 10.1007/s00253-023-12907-4 (PMC10774165; doi:10.1007/s00253-023-12907-4)
Supplement: Supplementary file 1 — Supplementary file1 (PDF 1250 KB) [file 253_2023_12907_MOESM1_ESM.pdf]

Applied Microbiology and Biotechnology

Correlating sugar transporter expression and activities to identify transporters for an orphan sugar substrate

Elisabeth Tamayo*, Basant Nada, Isabell Hafermann, J. Philipp Benz.

Fungal Biotechnology in Wood Science, Holzforschung München, TUM School of Life Sciences, Technical University of Munich, Freising, Germany.

*Corresponding author: tamayo@hfm.tum.de

Supplemental tables

Table S1. Oligonucleotides used in this study

Primer	Sequence 5' - 3'	Application
FW_pHTX7_cdt1	AAACACAAAAACAAAAAGTTTTTTTAATTTTAATCAAAAAATGTCGTCTCACGGCTCC	Cloning of *cdt-1*
RV_tCYC1_cdt1	GAGGGCGTGAATGTAAGCGTGACATAACTAATTACATGAAGCAACGATAGCTTCGGACAC	Cloning of *cdt-1*
FW_pHXT7_cdt2	ACACAAAAACAAAAAGTTTTTTTAATTTTAATCAAAAAATGGGCATCTTCAACAAGAAGC	Cloning of *cdt-2*
RV_tCYC1_cdt2	GAGGGCGTGAATGTAAGCGTGACATAACTAATTACATGATCAAGCAACAGACTTGCCCTC	Cloning of *cdt-2*
FW_pHTX7_809	AAACACAAAAACAAAAAGTTTTTTTAATTTTAATCAAAAAATGGCTCACAGCATAAACG	Cloning of *NCU00809*
RV_tCYC1_809	TTACATGACTCGAGGTCGACGGTATCGATAAGCTTCTAAATTGTAACTTTCTCGTCATCC	Cloning of *NCU00809*
cdt1_seq	GTTCGGAGGTTCGATTCC	Sequencing of *cdt-1*
809_seq	GCTCTATTATCGCTTCGTGG	Sequencing of *NCU00809*
cPCR_HXT7p	CAAGAACAAACAAGCTCAAC	Colony PCR
cPCR_tCYC1	ACCTAGACTTCAGGTTGTC	Colony PCR
qRTPCR_actin_FW	GTCTCTCCGACCGTATGCAG	RT-qPCR of *act*
qRTPCR_actin_RV	GCGAGAATGGAACCACCGAT	RT-qPCR of *act*
810_Fw_qPCR	CTGGCTCGAGGATTACGAAG	RT-qPCR of *NCU00810*
810_Rv_qPCR	TTCCATCAGGGTCTTCTTTCC	RT-qPCR of *NCU00810*

Table S2. List of 44 putative sugar transporters in *N. crassa*

Locus	TC category	Homology by phylogeny	Induction condition*	Protein length (aa)
NCU00450	2.A.2	Disaccharide transporter	Mannose	674
NCU00801	2.A.1.1	Cellodextrin transporter	Cellobiose	579
NCU00809	2.A.1.1	Disaccharide transporter	Arabinose	547
NCU00821	2.A.1.1	Pentose transporter	Fructose	562
NCU00988	2.A.1.1	Galacturonic acid transporter	Galacturonic acid	537
NCU01132	2.A.1.1	Pentose transporter	Arabinose	553
NCU01494	2.A.1.1	Polyol transporter	Xylose	674
NCU01633	2.A.1.1	Glucose transporter	Cellobiose	532
NCU01813	2.A.1.1	Pentose transporter	Mannose	565
NCU01868	2.A.1.1	Disaccharide transporter	Arabinose	523
NCU02188	2.A.1.1	Pentose transporter	Arabinose	518
NCU02582	2.A.1.1	Glucose transporter	Fucose	594
NCU03468	2.A.1.7	Hypothetical protein	Rhamnose	531
NCU04310	2.A.1.7	Fucose permease	Fructose	1050
NCU04537	2.A.1.1	Monosaccharide transporter	Maltose	539
NCU04963	2.A.1.1	Glucose transporter	Maltose	527
NCU05350	2.A.1.1	Disaccharide transporter	Xylose	498
NCU05394	2.A.1.7	Hypothetical protein	Fucose	548
NCU05585	2.A.1.1	Quinate transporter	Rhamnose	565
NCU05597	2.A.1.1	Hypothetical protein	Arabinose	520
NCU05627	2.A.1.1	Pentose transporter	Cellobiose	548
NCU05853	2.A.1.1	Cellobionic acid transporter	Cellobiose	541
NCU05897	2.A.1.7	Fucose permease	Cellobiose	471
NCU06026	2.A.1.1	Quinic acid transporter	Fructose	537
NCU06138	2.A.1.1	Pentose transporter	Xylose	583
NCU06358	2.A.1.1	Glucose transporter	Ribose	610
NCU06384	2.A.1.1	Pentose transporter	Fucose	532
NCU06522	2.A.1.1	Disaccharide transporter	Mannose	560
NCU06846	2.A.1.1	Myo-inositol transporter	Fucose	537
NCU07054	2.A.1.1	Disaccharide transporter	Arabinose	565
NCU07169	2.A.1.1	Hypothetical protein	Fucose	523
NCU07199	2.A.1.1	Cellobionic acid transporter	Fucose	520
NCU07607	2.A.1.1	Hypothetical protein	Fructose	557
NCU07861	2.A.1.1	Disaccharide transporter	-	597
NCU08114	2.A.1.1	Cellodextrin transporter	Cellobiose	525
NCU08152	2.A.1.1	Pentose transporter	Arabinose	537
NCU08180	2.A.1.1	Monosaccharide transporter	-	643
NCU08858	2.A.1.1	Disaccharide transporter	Ribose	581
NCU09287	2.A.1.1	Polyol transporter	Rhamnose	655
NCU09321	2.A.2	Disaccharide transporter	Ribose	550
NCU09358	2.A.1.1	Pentose transporter	Mannose	520
NCU10021	2.A.1.1	Glucose transporter	Cellobiose	553
NCU11342	2.A.1.1	Disaccharide transporter	Mannose	460
NCU12154	2.A.1.1	Disaccharide transporter	Galacturonic acid	424

* According to Wu et al. (2020). Condition for maximum induction is indicated.

Table S3. List of characterized sugar transporters in *N. crassa*

Protein name	Gene name	Locus	Function	Reference(s)
GLT-1	*sut-5*	NCU01633	D-Glucose transport	Li et al. (2014); Wang et al. (2017)
HGT-1	*hgt-1*	NCU10021	D-Glucose high-affinity transport	Xie et al. (2004); Wang et al. (2017)
HGT-2	*sut-9*	NCU04963	D-Glucose high-affinity transport	Du et al. (2010); Li et al. (2014); Wang et al. (2017)
RCO-3	*sor-4*	NCU02582	D-Glucose sensor	Madi et al. (1997)
LAT-1	*lat-1*	NCU02188	L-Arabinose (galactose, glucose, fructose) transport	Benz et al. (2014a); Li et al. (2015)
XAT-1	*sut-27*	NCU01132	L-Arabinose and D-xylose transport	Li et al. (2014)
XYT-1	*sut-7*	NCU05627	D-Xylose transport	Li et al. (2014)
AN25	*sut-15*	NCU00821	D-Xylose transport	Du et al. (2010)
GAT-1	*mfs-11*	NCU00988	D-Galacturonic acid and D-glucuronic acid transport	Benz et al. (2014b)
Qa-Y	*qa-Y*	NCU06026	Quinic acid transport	Case et al. (1992)
FRT-1	*sut-28*	NCU05897	L-Rhamnose and fucose transport	Wu et al. (2020)
CBT-1	*sut-12*	NCU05853	Cellobionic acid transport	Li et al. (2015); Cai et al. (2015)
CDT-1	*cdt-1*	NCU00801	Cellobiose, mannobiose, lactose, cellotriose and cellotetraose transport; transceptor function	Galazka et al. (2010); Li et al. (2015); Znameroski et al. (2014); Hassan et al. (2019)
CDT-2	*cdt-2*	NCU08114	Cellobiose, xylobiose (mannobiose), xylotriose and cellotriose transport; transceptor function	Galazka et al. (2010); Li et al. (2015); Cai et al. (2014); Znameroski et al. (2014); Hassan et al. (2019)

Supplemental figures

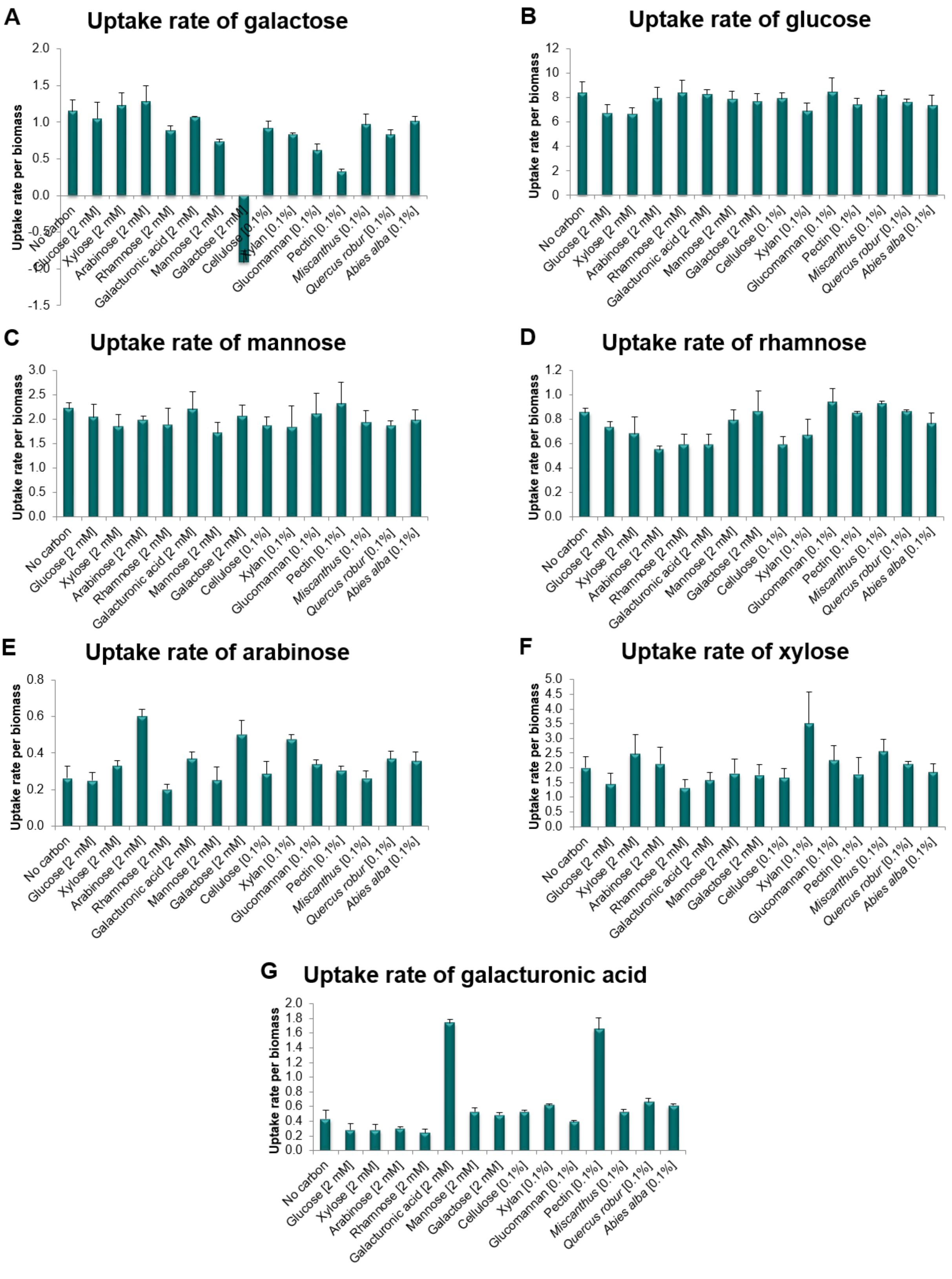

Fig. S1. Uptake rates of monosaccharides under different induction conditions. The uptake after 15 minutes of the four hexoses (A-D) D-galactose, D-glucose, D-mannose and L-rhamnose, the two pentoses (E-F) L-arabinose and D-xylose and one uronic acid (D-galacturonic acid, G) was measured in

the WT strain after induction under fifteen different conditions. Data are mean of three biological replicates. Error bars represent standard deviation.

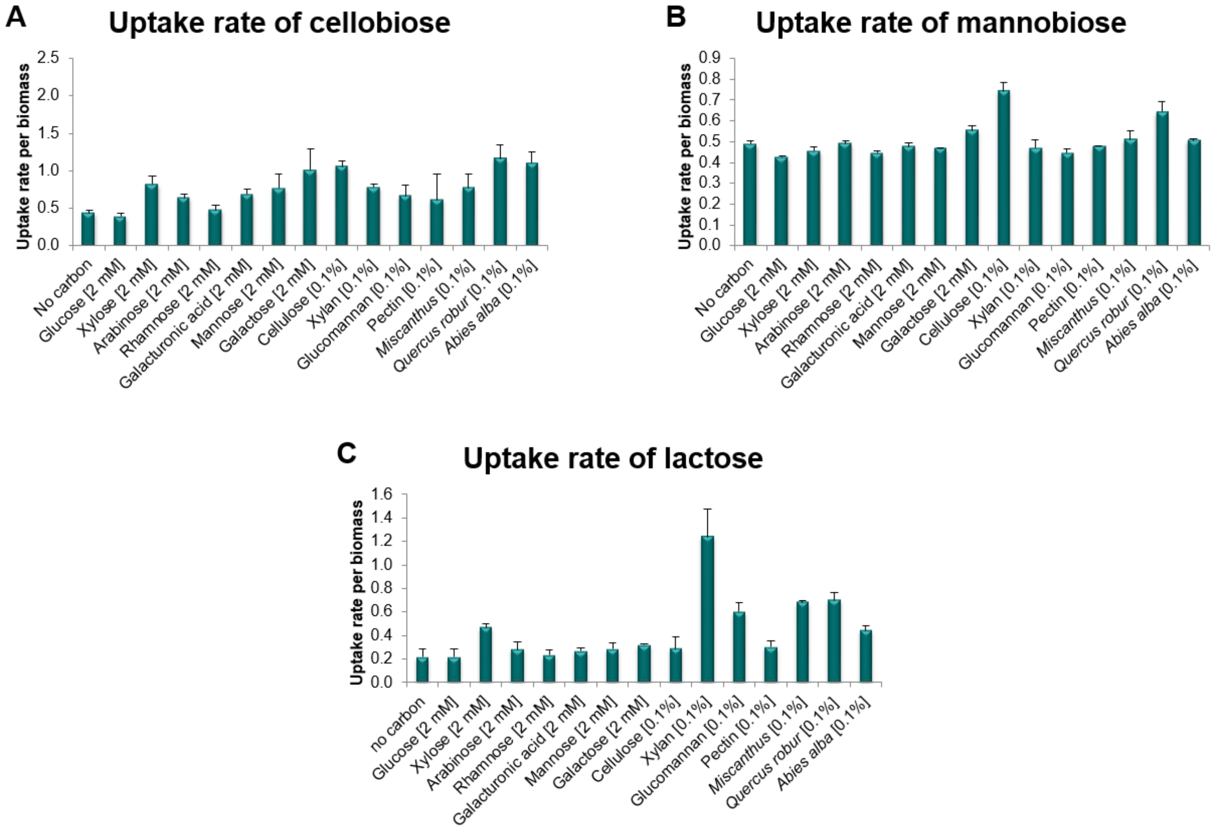

Fig. S2. Uptake rates of disaccharides under different induction conditions. The uptake after 15 minutes of the three disaccharides cellobiose (A), mannobiose (B) and lactose (C) was measured in the WT strain after induction under fifteen different conditions. Data are mean of three biological replicates. Error bars represent standard deviation.

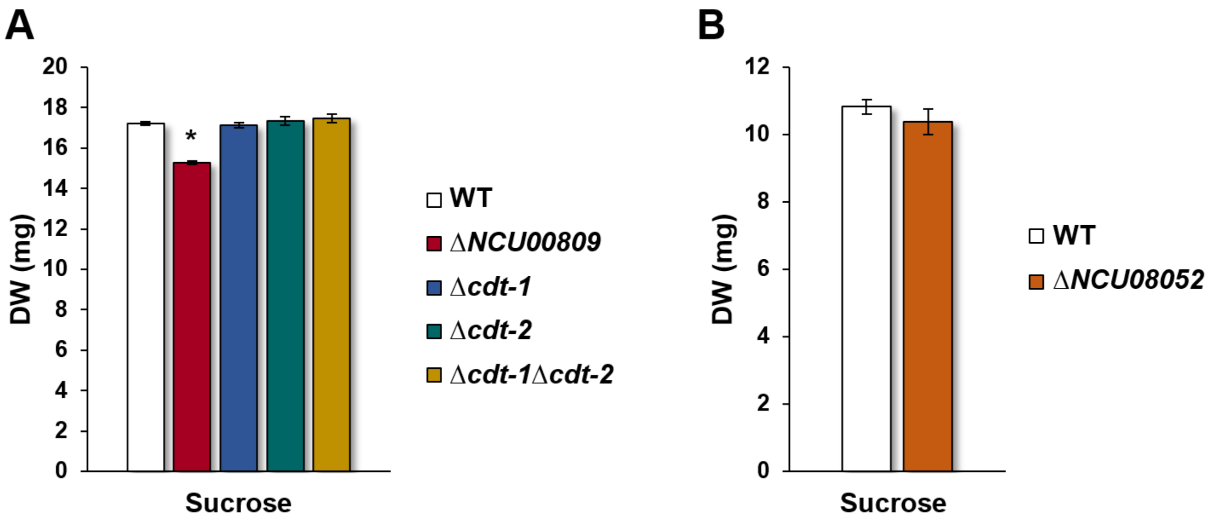

Fig. S3. Control growth assays of different *Neurospora crassa* mutant strains in sucrose media. A. Growth of different lactose mutants on MM + 1% sucrose as sole carbon source for 48 hours (n=4). **B.** Growth of Δ*NCU08052* mutant on MM + 1% sucrose as sole carbon source for 36 hours (n=4). Asterisks indicate statistical significance compared to the WT strain, *p <0.05. DW, dry weight.

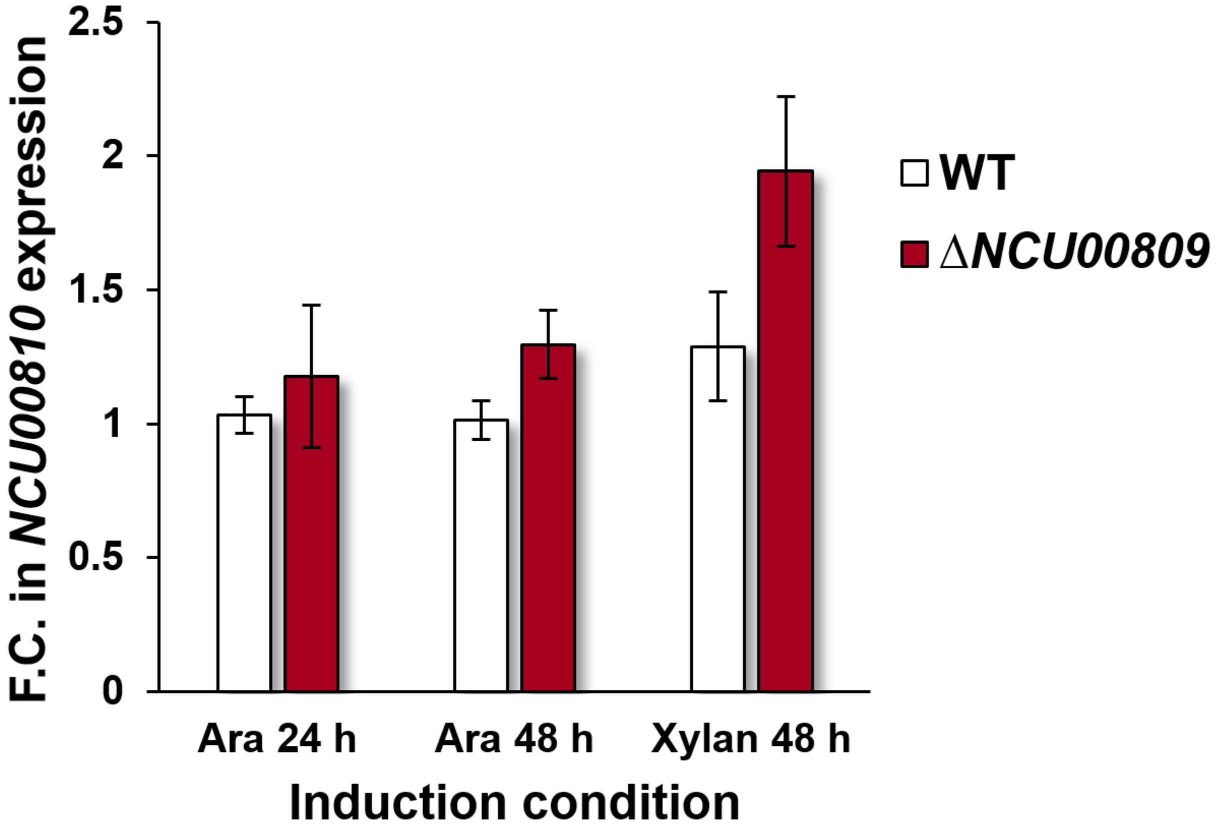

Fig. S4. Effect of *NCU00809* deletion on the expression of the β-galactosidase gene *NCU00810*. Fold change (F.C.) in expression was assessed after induction in 0.5% arabinose (24 h or 48 h) or 0.5% xylan (48 h). Data were normalized using the housekeeping gene *act* and with respect to WT values in the arabinose induction condition after 24 h. Relative expression levels were calculated by the $2^{-\Delta\Delta CT}$ method.

Data are mean ± standard error. Asterisks show statistically significant differences ($p < 0.05$) compared to the expression value in the WT strain.

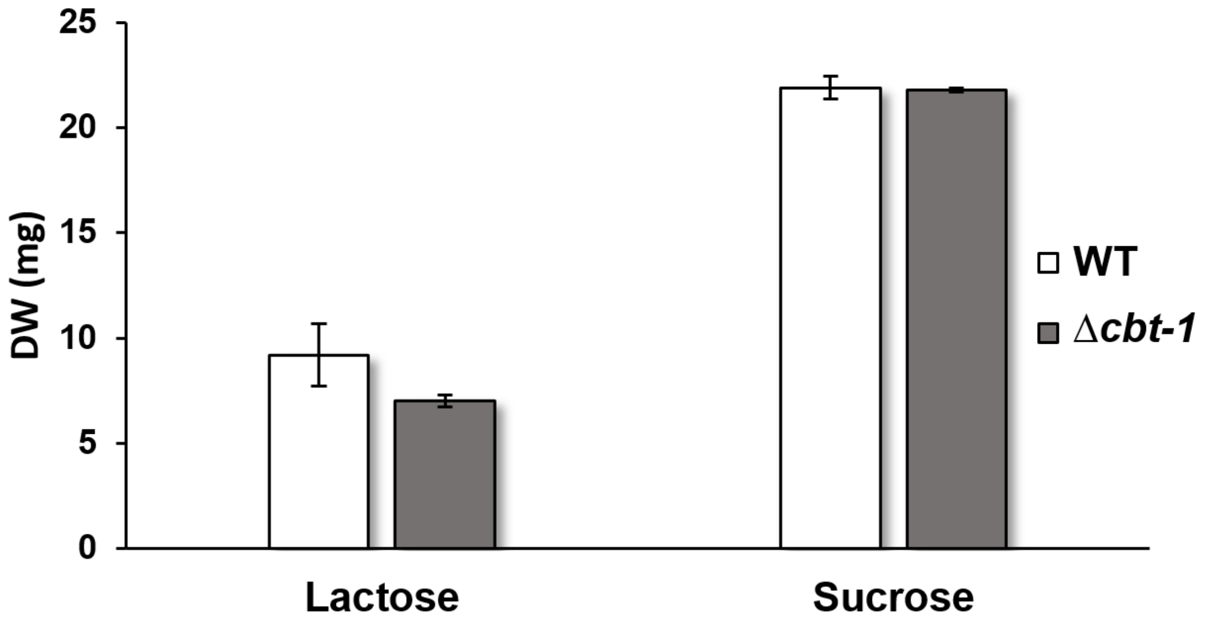

Fig. S5. Growth on lactose of the *Neurospora crassa* Δ*cbt-1* mutant strain. The Δ*cbt-1* mutant strain and the wild-type strain were grown on MM + 1% lactose (left) and on MM + 2% sucrose (right; control growth) as the sole carbon source for 6 and 2 days, respectively (n=4). DW, dry weight.

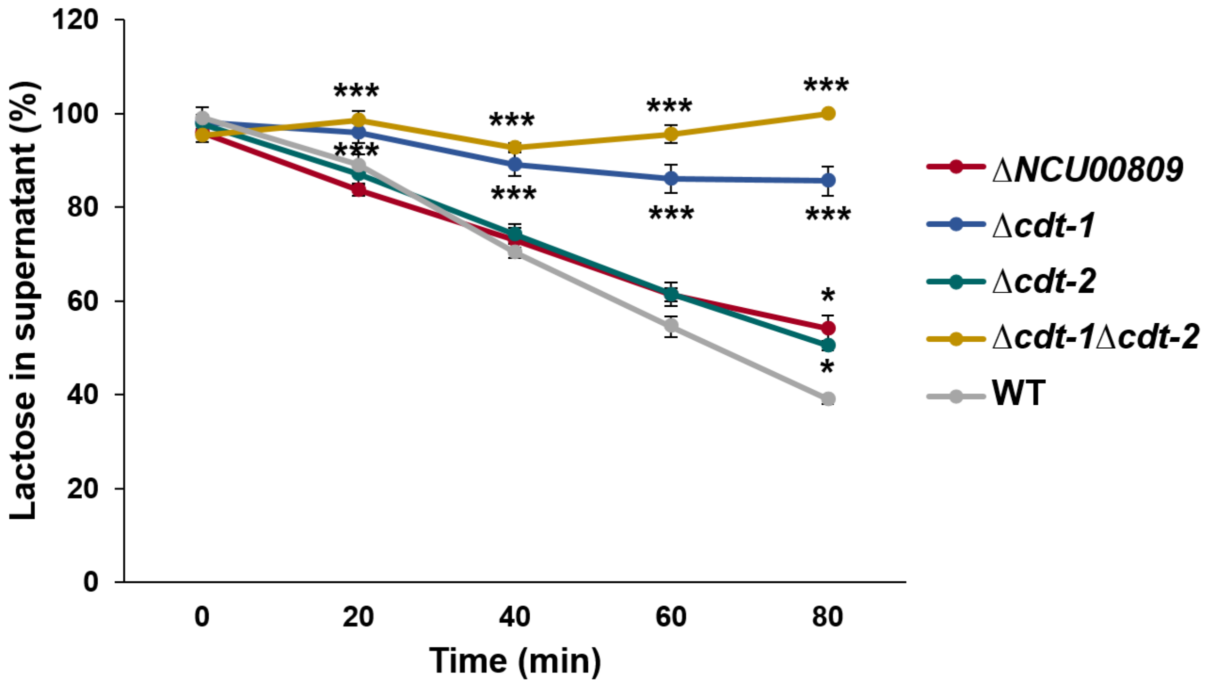

Fig. S6. Lactose uptake of different *Neurospora crassa* mutant strains in genes with homology to lactose transporters after lactose induction. Asterisks indicate statistical significance compared to the WT strain (n=3), *$p < 0.05$; ***$p < 0.001$.

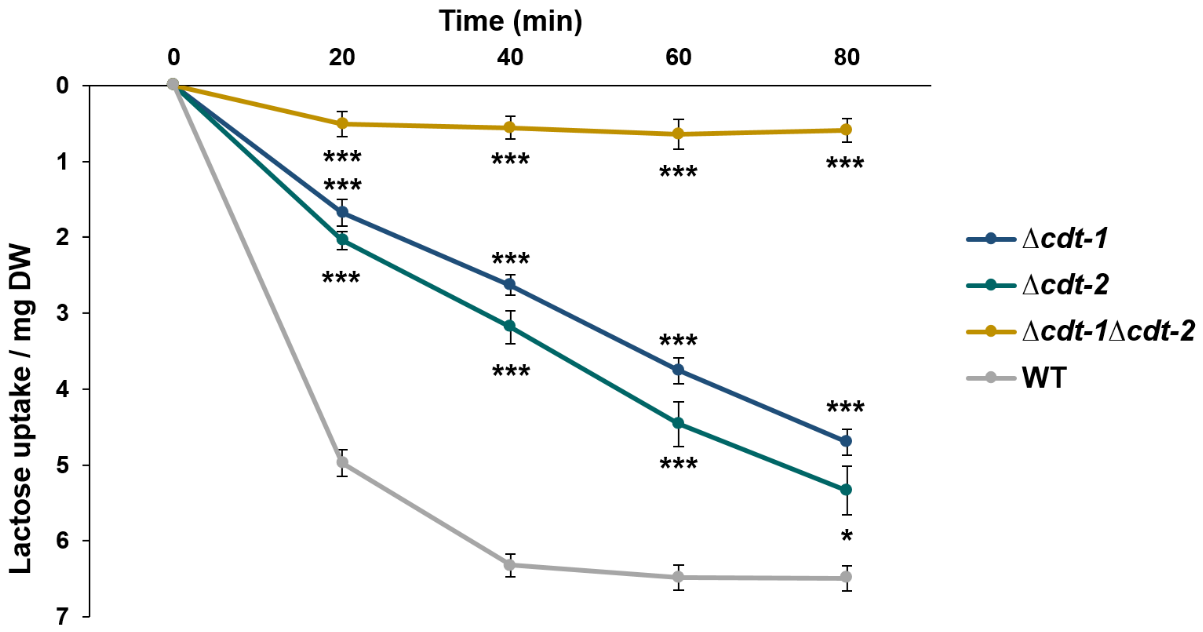

Fig. S7. Lactose uptake of different *Neurospora crassa* mutant strains in genes with homology to lactose transporters after cellulose induction. Asterisks indicate statistical significance compared to the WT strain (n=3), *$p < 0.05$; ***$p < 0.001$. DW, dry weight.

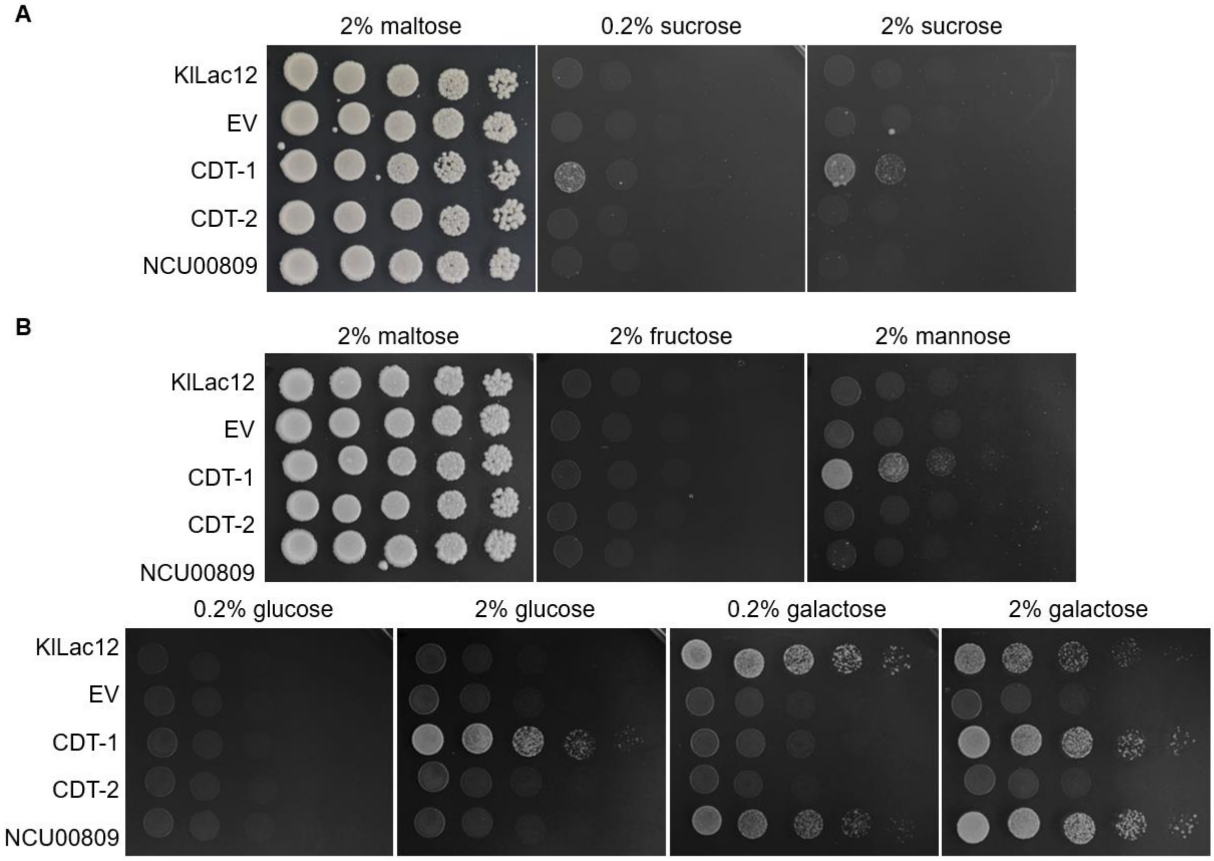

Fig. S8. Growth properties of the yeast strain EBY.VW4000 strain expressing *Neurospora crassa* CDT-1, CDT-2 or NCU00809. Strains were spotted on SC medium without uracil and leucine supplemented with 2% maltose (growth control) or with different sugars. **A.** Growth on 0.2% and 2% sucrose. Plates were incubated at 30° C for 7 days. **B.** Growth on 2% fructose, 2% mannose (top row); 0.2% glucose, 2% glucose, 0.2% galactose and 2% galactose (bottom row). Plates were incubated at 30° C for 3 days. The permease Lac12 from *Kluyveromyces lactis* (KlLac12) was included as a positive control for galactose transport (Rigamonte et al. 2011).

Supplemental references

Benz JP, Chau BH, Zheng D, Bauer S, Glass NL, Somerville CR (2014a) A comparative systems analysis of polysaccharide-elicited responses in *Neurospora crassa* reveals carbon source-specific cellular adaptations. Mol Microbiol 91(2):275-299. doi: 10.1111/mmi.12459

Benz JP, Protzko RJ, Andrich JM, Bauer S, Dueber JE, Somerville CR (2014b) Identification and characterization of a galacturonic acid transporter from *Neurospora crassa* and its application for *Saccharomyces cerevisiae* fermentation processes. Biotechnol Biofuels 7(1):20. doi: 10.1186/1754-6834-7-20. Erratum in: Biotechnol Biofuels (2017) 10:287.

Cai P, Gu R, Wang B, Li J, Wan L, Tian C, Ma Y (2014) Evidence of a critical role for cellodextrin transporter 2 (CDT-2) in both cellulose and hemicellulose degradation and utilization in *Neurospora crassa*. PLoS One 9(2):e89330. doi: 10.1371/journal.pone.0089330

Cai P, Wang B, Ji J, Jiang Y, Wan L, Tian C, Ma Y (2015) The putative cellodextrin transporter-like protein CLP1 is involved in cellulase induction in *Neurospora crassa*. J Biol Chem 290(2):788-796. doi: 10.1074/jbc.M114.609875

Case ME, Geever RF, Asch DK (1992) Use of gene replacement transformation to elucidate gene function in the *qa* gene cluster of *Neurospora crassa*. Genetics 130(4):729-736. doi: 10.1093/genetics/130.4.729

Du J, Li S, Zhao H (2010) Discovery and characterization of novel D-xylose-specific transporters from *Neurospora crassa* and *Pichia stipitis*. Mol Biosyst 6(11):2150-2156. doi: 10.1039/c0mb00007h

Galazka JM, Tian C, Beeson WT, Martinez B, Glass NL, Cate JH (2010) Cellodextrin transport in yeast for improved biofuel production. Science 330(6000):84-86. doi: 10.1126/science.1192838

Hassan L, Lin L, Sorek H, Sperl LE, Goudoulas T, Hagn F, Germann N, Tian C, Benz JP (2019) Crosstalk of cellulose and mannan perception pathways leads to inhibition of cellulase production in several filamentous fungi. mBio 10(4):e00277-19. doi: 10.1128/mBio.00277-19

Li J, Lin L, Li H, Tian C, Ma Y (2014) Transcriptional comparison of the filamentous fungus *Neurospora crassa* growing on three major monosaccharides D-glucose, D-xylose and D-arabinose. Biotechnol Biofuels 7(1):31. doi: 10.1186/1754-6834-7-31

Li X, Chomvong K, Yu VY, Liang JM, Lin Y, Cate JHD (2015) Cellobionic acid utilization: from *Neurospora crassa* to *Saccharomyces cerevisiae*. Biotechnol Biofuels 8:120. doi: 10.1186/s13068-015-0303-2

Madi L, McBride SA, Bailey LA, Ebbole DJ (1997) *rco-3*, a gene involved in glucose transport and conidiation in *Neurospora crassa*. Genetics 146(2):499-508. doi: 10.1093/genetics/146.2.499

Rigamonte TA, Silveira WB, Fietto LG, Castro IM, Breunig KD, Passos FM (2011) Restricted sugar uptake by sugar-induced internalization of the yeast lactose/galactose permease Lac12. FEMS Yeast Res 11(3):243-251. doi: 10.1111/j.1567-1364.2010.00709.x

Wang B, Li J, Gao J, Cai P, Han X, Tian C (2017) Identification and characterization of the glucose dual-affinity transport system in *Neurospora crassa*: pleiotropic roles in nutrient transport, signaling, and carbon catabolite repression. Biotechnol Biofuels 10:17. doi: 10.1186/s13068-017-0705-4

Wu VW, Thieme N, Huberman LB, Dietschmann A, Kowbel DJ, Lee J, Calhoun S, Singan VR, Lipzen A, Xiong Y, Monti R, Blow MJ, O'Malley RC, Grigoriev IV, Benz JP, Glass NL (2020) The regulatory and transcriptional landscape associated with carbon utilization in a filamentous fungus. Proc Natl Acad Sci U S A 117(11):6003-6013. doi: 10.1073/pnas.1915611117

Xie X, Wilkinson HH, Correa A, Lewis ZA, Bell-Pedersen D, Ebbole DJ (2004) Transcriptional response to glucose starvation and functional analysis of a glucose transporter of *Neurospora crassa*. Fungal Genet Biol 41(12):1104-1119. doi: 10.1016/j.fgb.2004.08.009

Znameroski EA, Li X, Tsai JC, Galazka JM, Glass NL, Cate JH (2014) Evidence for transceptor function of cellodextrin transporters in *Neurospora crassa*. J Biol Chem 289(5):2610–2619. doi: 10.1074/jbc.M113.533273
